# Patients' Health & Well-Being in Inpatient Mental Health-Care Facilities: A Systematic Review

**DOI:** 10.3389/fpsyt.2021.758039

**Published:** 2022-01-03

**Authors:** Clara Weber, Virna Monero Flores, Theresa Poppy Wheele, Elke Miedema, Emma Victoria White

**Affiliations:** ^1^Institute of Facility Management, Life Sciences and Facility Management, Zurich University of Applied Sciences, Zurich, Switzerland; ^2^Environmental Psychology Department, School of Psychology, University of Surrey, Guildford, United Kingdom; ^3^Architectural Theory and Methods, Department of Architecture and Civil Engineering, Chalmers University of Technology, Gothenburg, Sweden

**Keywords:** healing environments, systematic review, well-being, mental health, recovery outcomes, psychiatric hospital

## Abstract

**Background:** Previous research indicates that the physical environment of healthcare facilities plays an important role in the health, well-being, and recovery outcomes of patients. However, prior works on mental healthcare facilities have incorporated physical environment effects from general healthcare settings and patient groups, which cannot be readily transferred to mental healthcare settings or its patients. There appears to be a specific need for evidence synthesis of physical environmental effects in mental healthcare settings by psychopathology.

**Purpose:** This review evaluates the state (in terms of extent, nature and quality) of the current empirical evidence of physical environmental on mental health, well-being, and recovery outcomes in mental healthcare inpatients by psychopathology.

**Method:** A systematic review (PRISMA guidelines) was performed of studies published in English, German, Dutch, Swedish, and Spanish, of all available years until September 2020, searched in Cochrane, Ovid Index, PsycINFO, PubMed, and Web of Science and identified through extensive hand-picking. Inclusion criteria were: Adult patients being treated for mental ill-health (common mental health and mood disorders, Cochrane frame); inpatient mental health care facilities; specifications of the physical and socio-physical environment (e.g., design features, ambient conditions, privacy); all types of empirical study designs. Quality assessment and data synthesis were undertaken.

**Results:** The search retrieved 1,068 titles of which 26 met the inclusion criteria. Findings suggest that there is only indicative evidence of the impact of the physical healthcare environment on patients' mental health, well-being, and recovery outcomes. There is significant lack of pathology-specific evidence. Methodological shortcomings and empirical scarcity account for the poor evidence.

**Conclusion:** This review highlights the need for more research using advanced study designs.

## Introduction

Treatment and recovery in mental health-care facilities has long been acknowledged to go beyond immediate therapeutic and pharmacological interventions. Early salutogenesis and milieu studies postulated a necessary focus on health protection and promotion as opposed to diagnosis and treatment of illness, and an incorporation of the broader psychological impact of the hospital environment upon both staff and patients e.g., (1–4). Based on early evidence, such proponents argue that the course of treatment in mental health-care is largely affected by the organization and characteristics of the broader physical and social environment of the facility (5). In recent times, salutogenic and milieu approaches to healthcare management and building design have been recommended for different types of healthcare facilities and patient populations (6–9). Design has become integral to quality healthcare (10, 11) and part of a value-centered system, enabling effective care as “tool and healer” (12), pp. 670–671. The architecture profession and building industry have also started to acknowledge architecture's therapeutic potential and risks (13–17).

Although awareness of the relationship between the quality of the physical environment and healthcare delivery and recovery has increased over recent decades, heuristic guidance documents and frameworks for mental healthcare facilities, or the study thereof, tend to incorporate evidence of the effects of the physical environment on patients with *physiological* ill-health [e.g., (18–25)]. This is problematic, since the evidence from generic healthcare settings of treating patients with physiological ill-health has been considered poor (12, 26). We also argue that the findings in these healthcare contexts and patient groups cannot be readily transferred to mental healthcare settings. Treatment and recovery specifications, which are specific to mental health-care settings and affect the organization, design, and equipment of the physical environment, include designs to: prevent self-inflected injuries and suicidality; customize varying levels of safeguarding (e.g., observation by night, fixation, seclusion, restriction of freedom of movement) (21); and address residential/housing needs (e.g., psychological needs for safety/security, territoriality, privacy, appropriation, familiarity, socializing, belonging/attachment) (21, 27–29). The latter point is of particular significance to mental healthcare settings (when compared to generic healthcare settings), given the patients' longer hospitalization duration (the setting becomes a temporary home), the larger radius of movement within the facility (more physiological capabilities) (21, 29), and the recovery aim of regaining everyday competencies to lead a healthy independent life after hospitalization (13, 19). Further, we argue that evidence cannot be readily transferred because of pathology specifications. Patients with psychopathologies are considered particularly vulnerable to environmental influences due to perceptual variation and sensory processing differences, which are associated with certain psychopathologies (e.g., acute psychosis, agnosia, dementia, schizophrenia, depression, manic syndromes, anxiety disorders) and can result in hyper- or hyposensitivity toward environmental stimuli (4, 12, 13, 21, 29–36).

Within the field, there also appears to be no synthesis of the available evidence of physical environmental effects in mental healthcare settings on specific psychopathologies. Evidence synthesis can aid in the identification of research gaps, as well as guiding development to improve mental healthcare facilities, which still fall short in their health-promotive approach compared with general care facilities (37). Specifically, evidence synthesis will aid the development of evidence-based design guidance on the requirements for special psychiatric pathologies, which is currently overdue (23, 31). Further, available best practice guidelines for designing psychiatric inpatient facilities [e.g., (38, 39)] are “based mainly on clinical conjecture, professional experience, and anecdote” [(23), p.2]. Overall, the psychopathology-specific evidence-base of the positive and negative influence of the physical environment on mental health patients' health, well-being, and recovery outcomes remains unclear. This systematic review therefore has the following objective:

Clarify the state (in terms of extent, nature and quality) of the current empirical evidence of the physical environmental on mental health, well-being, and recovery outcomes in mental healthcare inpatients by psychopathology.

## Methods

A search was carried out based upon PRISMA guidance (40), which aimed to locate all relevant empirical, peer-reviewed evidence for the relationships between physical and socio-physical environmental characteristics of inpatient mental health-care facilities and mental health, well-being, and recovery outcomes in inpatients.

### Eligibility Criteria

Papers were eligible for inclusion if they examined/included one of the following: adult patients being treated for mental ill-health (common mental health and mood disorders, according to Cochrane framework of common mental health disorders); inpatient mental health care facilities as the study context; the built environment as a study object; the study of any specifications of the physical and socio-physical environment (e.g., design features, ambient conditions, privacy) and the health and well-being effects in patients. Papers were included from all available years until September 2020, with all types of study designs, published in English, German, Spanish and Dutch.

Exclusion criteria were: studies of forensic facilities^1^ or ambulant/day-care facilities; patients being treated for non-psychological pathologies (e.g., labor, surgery, heart disease) or pathologies not listed in the Cochrane Framework of Common Mental Health Disorders (e.g., gerontopsychiatric pathologies such as dementia); studies using participants under the age of 18 years; a focus on environmental infrastructure or design principles for safety and suicide prevention, due to its highly regulated nature; examination of the psychosocial and procedural characteristics of the environment (e.g., Ward Atmosphere Scale); studies not conducted in the field or lab, such as expert interviews (health care staff, design consultants); unspecific analysis procedures, where the relative effects of environmental characteristics are non-identifiable (e.g., use of composite variables clustering together physical/socio-environmental variables with psychosocial variables); outcomes not related to mental health, well-being or recovery related (e.g., procedural efficiency); associated peer-reviewed papers of meta-analyses, dissertations/theses, case studies, and conference papers were excluded; systematic literature reviews were excluded but used for handpicking.

### Search Strategy

Due to the small evidence base on the topic, the search strategy was not devised using a PICOS or similar grid. To avoid unduly narrowing the search and excluding relevant text, the search strategy only specified the following search terms/subjects: (S1) general mental healthcare conditions (psychot^*^, psychos^*^, psychiat^*^, neurotic^*^, mental) and possible treated psychopathologies (based on Cochrane Framework of Common Mental Health Disorders); (S2) the setting as study context; (S3) the built environment as the object of the study; and (S4) specifications of the physical and socio-physical environment. The search term strategy and selection of S1 was guided and reviewed by an independent, established researcher in mental health sciences and nursing. Search terms S2-S4 were based on an extensive scoping review including key texts in the field [e.g., (6–9, 42–44)]. The final selection of S2–S4 terms was reviewed by a researcher of the group experienced in health architecture research (EM). Subject strings were tested for eligible study retrieval. This resulted in splitting the originally merged terms of S3 and S4 into two separate subjects. The subject strings were then combined in one search-string, including all four subjects at once, “S1 AND S2 AND S3 AND S4”, in the title and/or abstract (not in key words). The combined string was also tested for eligible study retrieval, with a sample of 25 papers was checked in each database. In terms of search limits, no specifications (such as time, language, publication types) were set, to maintain a wide search. Search operators were used to include different spellings (e.g., colour and color) and derivations of the same root word (e.g., health and healing). See Table 1 for subject terms.

**Table 1:** Subject terms.

**Subject**	**Description**	**Search terms**
S1	Psychopathologies: Specifies psychopathologies according to Cochrane Framework Of Common Mental Health Disorders	adjustment disorder^*^, affective disorder^*^, anxiety, **behavioral issues**, bipolar, conversion disorder^*^, depress^*^, eating disorder^*^, factitious disorder^*^, fatigue syndrome, mental, mood disorder^*^, neurotic^*^, obsessive compulsive disorder^*^, panic disorder^*^, personality disorder^*^, phobic disorder^*^, post traumatic stress disorder^*^, psychiat^*^, psychos^*^, psychosexual disorder^*^, psychot^*^, schizophreni^*^, seasonal affective disorder^*^, self injurious behavior^*^, somatoform disorder^*^
S2	Setting as study context: Specifies all types of in-patient clinical settings where the studies could take place	clinic^*^, facilit^*^, facility^*^, heal^*^, heal^*^, heal^*^ unit^*^, hospital, patient room, psychiatric wards, therapy room, ward^*^
S3	Setting as study object: Specifies settings and conceptual approaches	architectur^*^, built environment, building design, designed environment, heal^*^ heal^*^ design^*^, heal^*^ environment^*^, physical environment
S4	Physical or socio-spatial environmental characteristics	affordanc^*^, ambien^*^, ambient environment, annoy^*^, art, biophil^*^, colo?r, control^*^, crowd^*^, distract^*^, environment^*^ control, environment^*^ stimul^*^, green^*^, indoor environment^*^, interact^*^, interrupt^*^, light^*^, natur^*^, nois^*^, personal spac^*^, plant^*^, priva^*^, spat^*^, spac^*^, sound^*^, temperature, territorial^*^, wayfind^*^

*Overarching search terms are marked bold*.

### Data Sources

The following databases were used: **Web of Science Core Collection** (1956–2020), **PubMed** (1788–2020), **PsychINFO** (1803–2020), **Ovid Index** [The Psychiatric Interview: Practical Guides in Psychiatry 1965–2020; Journals@Ovid 1860–2020; PsycARTICLES & psyCRITIQUES (1860–2020); APA PsycArticles (1860–2020); APA PsycExtra (1908–2020); APA PsycTests (1910–2020); PSYNDEXplus Literature and Audiovisual Media (1977–2020); PSYNDEXplus Tests (1945–2020); Ovid Emcare (1995–2020); Ovid Nursing Database (1946–2020)] and **Cochrane** (1946–2020). These databases are commonly used in healthcare building design literature reviews as they provide both sources from health, design and engineering sciences (11, 45–48)^2^. Extensive handpicking was informed by retrieved and subsequently excluded literature reviews and other types of studies (e.g., systematic literature reviews). Additionally, three titles (49–51) were sourced through *post-hoc* expert recommendation (reviewer).

### Conducting Searches

**Figure 1:**
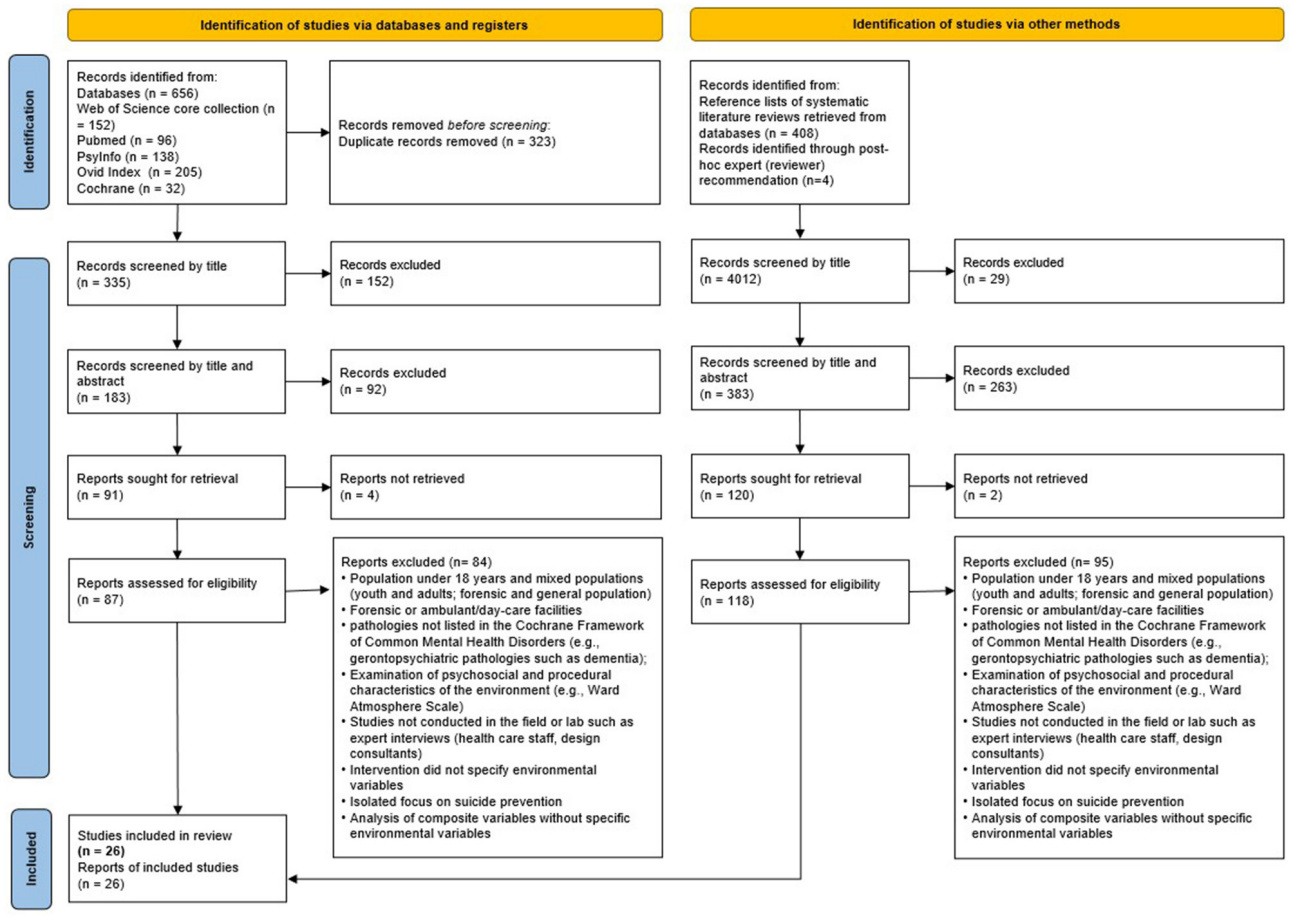
Prisma flow chart.

One researcher (VMF) conducted the search between June and July in 2020. The search identified 656 records, which included at least one term of each search string in the title or in the abstract (see Figure 1). The search retrieved non-eligible studies [e.g., (16) systematic reviews], which were excluded but informed handpicking. Handpicking resulted in 408 additional titles; *post-hoc* expert recommendation (reviewer) resulted in three additional titles (49–51). After duplicates were removed, a team of six researchers (CW, VMF, EM, EH, MJ, PBG) assessed the eligibility of studies in a four-step process: (1) screening titles, (2) screening abstracts, (3) full text skimming, and (4) full text review and application of exclusion-inclusion criteria (following PRISMA guidance). At each screening step, items were assigned to a different member. Uncertainties at each screening stage were discussed within the whole group on a regular basis to achieve consensus. A total of 26 studies remained and were included in this review.

### Data Extraction

A standardized data extraction template was designed by one team member (CW) and included: study number, first author, year, country, reference number, study type (qualitative, quantitative, mixed-methods), target population (e.g., patients with particular psychopathologies), participants and sample size (e.g., nurses being interviewed on patients well-being), context/inpatient mental health-care facilities (number and type of facility/wards studied), study objectives, measures used (e.g., standardized scales), quality rating (overall rating of study's quality assessment), overall conclusions of the study, key findings pertinent to this review, building/design features (associated with patients' health and well-being), main and sub-domain of the effect of the environment (see framework 2.7.1), and outcomes (e.g., worsening of symptoms, aggression, length of stay) and their domains (mental health, well-being, and recovery outcomes)^3^. Data was extracted and reviewed by four researchers (CW, VMF, TPW, EM). An external researcher reviewed the data extraction for validity (EVW).

### Quality Assessment

To account for the heterogeneous nature of the included studies, the Mixed Methods Appraisal Tool (MMAT) (52) was used for study type categorization and quality assessment. The tool allows the appraisal of five study categories: qualitative research, randomized controlled trials, non-randomized studies, quantitative descriptive studies, and mixed-methods studies. Depending on study category, the appraisal tool considers: (1) method appropriateness, (2) analytical rigor, (3) bias, (4) confounding variables, and (5) reporting. As the number of rating questions vary by study category, the maximum overall score a study could obtain fell between 5 and 25. Since like-for-like comparisons were therefore challenging, a modification to the overall score format was employed. Overall scores were transformed into percentages to permit cross-category comparisons. Studies were classified as following: Excellent 100–85%, good 84–70%, fair 69–50%, and poor <49%. Quality ratings were not used as an exclusion criterion since the search objective was to include all available research. Studies rated as poor quality were therefore included in the review; quality ratings were not used as exclusion criteria as the search objective was to include all available research. Quality was assessed by four researchers (CW, VMF, EH, MJ) and reviewed by an external researcher for validity (EVW). Uncertainties were discussed within the whole group to achieve consensus.

### Data Analysis

Data was tabulated, categorized and synthesized narratively. Quantitative and qualitative meta-analyses were inappropriate analysis forms due the heterogeneous nature of the retrieved studies (53).

### Analysis Framework

The narrative analysis was rooted in (54) revised ecological model of Person-Environment (P-E) fit [cf. (55)], which has been used in various contexts and with different populations, including psychiatric institutions [e.g., (56–58)]. This ecological P-E fit model attempts to tease out the multi-tiered subjective dimensions of P-E fit on well-being (55). The model's ecological equation *[B* = f(P, E, P^*^E)] specifies that “behavior (*B*) is a function (*f* ) of the personal characteristics (*P*), [including personal resources and needs] and environmental characteristics (*E*) [including environmental resources and demands], together comprising a “subjective appraisal” by which the individual perceives the life condition not only through the present situation but through future expectations as well as through past experience” [(55), p.3]. The interaction term P^*^E represents P-E fit as congruence between personal needs with the environmental resources. Simply stated, behavior is a result of how the environment meets the needs of the individual. Well-being is therefore likely when there is adequate P-E fit. Lawton (59) specifies the environment as consisting of social and physical environmental dimensions, of which the latter differentiates between the objective measurable environment and the phenomenal environment. As such, this study understands the physical environment as offering demands, resources, affordances, and meanings. This understanding is further specified by frameworks of the psychiatric physical environment (4, 7, 19, 21) and takes an environmental psychological approach to clustering the evidence. The following analysis will therefore specify the evidence along four dimensions:

- Dimension (1), social stimulation in space, captures aspects that relate to regulating and reducing social interaction/demands afforded by the environment, and incorporates socio-environmental phenomena such as privacy, crowding, or personal space.^4^
- Dimension (2), environmental stimulation, has two levels. Firstly, it captures environmental stimuli that are demands and create sensory stress. Secondly, it captures environmental stimuli that are resources and can be positioned as therapeutic sensory stimulation.
- Dimension (3), environmental control, captures a sense of control afforded by the environment and is highly related to the other dimensions; it includes freedom of choice and behavioral independence, information access and control.
- Dimension (4), symbolism/associations, captures meanings of the environment such as home-like vs. institutional, and is strongly related to the normalization theory aiming for de-institutionalization of psychiatric settings (19).

### Specification of Outcomes

#### Recovery

Despite the paradigm shift toward promotion of patient-centered concepts of recovery [e.g., (60)], this review understands recovery solely from a traditional service-based, objective, or clinical recovery perspective (61) as of the immaturity of the research field. Service-based or clinical recovery is classically defined according to symptoms and various dimensions of functioning and is systematically assessed with clinicians' instruments (61). The review aimed to tease out studies that systematically test (with the use of clinical assessments) whether the institutional environment improves/hastens recovery (improvement of symptoms and functioning) or impedes/slows recovery (worsening of symptoms and functioning). If studies did not assess the effect of the institutional environment on clinical recovery directly, proxy measures, such as length of stay, were considered recovery outcomes.

#### Mental Health

In this review, mental health is approached from a subjective, patient perspective and includes any non-systematic, *ad-hoc* assessment (quotes by healthcare staff) and self-reports of customers/users/patients (e.g., focus groups, patient interviews) on symptoms, psychotic behavior, functional deficits, and states of significant, persistent emotional distress.^5^

#### Well-Being

This review understands well-being as a combination of a subjective, hedonic perspective, and a psychological, eudaimonic perspective [(64, 65) for in-depth discussion]. Subjective wellbeing includes, according to common assessment strategies, global life satisfaction or positive/negative affect (66, 67). Psychological well-being concerns the development of self-potential (68). According to the conceptualization of Ryff (68), its assessment includes autonomy, environmental mastery, personal growth, positive relations with others, purpose in life, and self-acceptance (69). Aspects of environmental comfort/discomfort were also considered as well-being.

## Results

### Study Characteristics

The full data extraction table can be found in the Supplementary Table 2 and a summary of the main study characteristics found below, in Table 2. The following text lists individual study identifiers where there is additional information to the date extraction table.

**Table 2:** Results.

**Author/year/** **country /reference no**.	**Study type**	**Target population**	**Participants & sample size**	**Methods/Measure**	**Qual**	**Building/design feature**	**Domain**	**Outcome**
Ben-Zeev et al., 2017, USA (70)	Quantitative non-randomized (cross-sectional)	Patients with high risk of violence (schizophrenia, schizoaffective d., bipolar d., co-occurring substance use d., violence-related incidences)	Patients (schizophrenia, schizoaffective d., bipolar d.) (*n =* 27)	Speech duration, movement & patient location (via smartphone sensors & beacons, mHealth); Diary study (questionnaires, 6-times/day)	60%	Noise	ES	Well-being (violent ideation)
Bowers et al., 2010, UK (71)	Quantitative (descriptive)	Patients (unspecified), staff & visitors	Total (*n =* 1227) - Patients (*n =* 393) - Staff (*n =* 638) - Visitors (*n =* 168)	Questionnaire (postal); frequency of ward door being locked; 34 Likert scaled items on acceptability of door locking an acute psychiatric inpatients ward (18 items effect on patients, seven items effect on staff, three items effects on visitors, six items ACMQ)	60%	Locked ward doors	EC S/A	Mental health (Increased adverse feelings: Depression/hopelessness, anger/frustration/irritation, feeling trapped/desperate to escape, worthless/rejected, hinted recovery impacts)
Brooks et al., 1994, USA (72)	Quantitative non-randomized (cross-sectional)	Patients (unspecified)	Measure at ward level (prevalence: bipolar d., manic type, paranoid schizophrenia, psychotic d., adjustment d.) (*n =* 6)	No. seclusion or restraint incidences (1 year); Patient census/unit at or over capacity	60%	Crowding/lack of space; <100 square feet (9 square meter)/patient	SS	Well-being (aggressive behavior via seclusion and constraint incidences)
Nanda et al., 2011, USA (73)	Mixed-methods, convergent design (quantitative non-randomized & qualitative descriptive)	Patients (unspecified)	Staff (*n =* 22) (patients unspecified, in crisis requiring hospitalization on acute unit)	PRN medication; Focus groups (staff)	47%	Artwork in patient lounge: 1) nature photograph, 2) landscape (abstract-representational), 3) chaotic abstract	ES S/A	Well-being (anxiety, agitation)
Ulrich et al., 2018, Sweden (22)	Quantitative non-randomized (cross-sectional)	Patients (unspecified)	Measurement at hospital level (across all: schizophrenia or other psychosis, bipolar d., personality d., suicide risk) (*n =* 3)	No. compulsory injections & restraints	80%	Single rooms, communal areas (spatial, adjust. furniture), low social density, noise reduction, control, garden, nature views, nature art, daylight, sightlines room-communal areas	SS ES EC	Well-being (aggressive behavior)
Bowers et al., 2009, UK (74)	Quantitative non-randomized (cross-sectional)	Patients with acute mental d.s (unspecified)	Measurement at ward level (*n =* 136)	Patient-staff Conflict Checklist (PCC-SR; staff)	40%	Locked ward doors	EC	Well-being (aggressive behavior, verbal aggression, physical aggression toward objects, physical aggression toward others)
Gallop et al., 1996, Canada (75)	Qualitative (descriptive)	Patients (female) with history of sexual and/or physical abuse	Female patients with sexual and/or physical abuse history (*n =* 18)	Semi-structured interviews	80%	Single vs. mixed-gender wards, soft lights at night (oppose to flashlight use), closed bedroom doors	SS EC	Mental health (trauma-related safety & control feelings)
Johnson and Delaney, 2006, USA (76)	Qualitative (grounded theory)	Patients (unspecified)	Patients (depression, schizophrenia, schizoaffective d., bipolar affective d.) (*n =* 12), Staff (*n =* 16)	Observations; Formal interviews (patient & staff)	100%	Crowding, personal space/common areas of adequate size, visibility (ward design & location nursing rooms), rules managing spaces & people accessibility (incl. personal space & territoriality), tangible boundaries (locked doors, closed-off areas e.g., kitchen)	SS EC	Well-being (Aggressive behavior)
Lindgren et al., 2015, Sweden (77)	Qualitative (phenomenological)	Patients (female) who self-harm	Female patients who self-harm (*n =* 6)	Observations; Informal interviews	100%	Crowding, locked ward doors, beds not in room & frequently moved, noise	SS ES EC	Mental health (trauma-related feelings of confusion and distress, states of panic, wish to escape, sleep disruption)
Beauchemin and Hays, 1996, Canada (91)	Quantitative non-randomized (quasi-experimental)	Patients with depression	Patients with depression (major depressive d. single/recurrent, bipolar, depression N.O.S.) (*n =* 174)	Admissions records (2 years)	60%	Natural light in patient rooms, either bright (max. 5000 lux) or dim (max. 300 lux)	ES	Recovery (length of stay)
Holmes et al., 2004, Canada (78)	Qualitative (phenomenological)	Patients (unspecified)	Patients with psychotic d.s (*n =* 6)	In-depth interviews	100%	Seclusion room	SS EC S/A	Well-being (feelings of exclusion, rejection, abandonment, anger, fear, shame, humiliation, sadness, depressive feelings)
Maloret and Scott, 2018, UK (79)	Qualitative (phenomenological)	Patients with autistic spectrum condition (ASC)	Former psychiatric inpatients with ASC diagnosis (co-diagnoses: anxiety, psychotic, mood d., depression, eating d., addiction) (=20)	Semi-structured interviews	80%	Bright lighting, air conditioning & other noise, strong smells (cleaning products), need for quiet and solitude space	ES	Mental health [anxiety and related coping strategies (e.g., aggression, self-harm, social withdrawal)]
O'Brien and Cole, 2004, Australia (80)	Mixed-methods, convergent design (quantitative descriptive & qualitative phenomenological)	Patients requiring close observation (e.g., suicidal patients, not specified)	Patients (who had been cared for in the close observation area, unspecified), relatives, staff (*n =* 42)	No. seclusion incidences & PRN medication use (1 month); Security use (5 months); Interviews & focus groups (patients, relatives, staff)	80%	Eight-bed close observation area in fish-bowl design (lack of privacy and doors, no environmental withdrawal possibilities given shared room), mixed-gender ward, prison-like atmosphere, poor environmental conditions (bathroom, toilets) and little comfort	SS ES EC S/A	Well-being (feeling unsafe, discomfort, feeling traumatized)
Benedetti et al., 2001, Italy (81)	Quantitative non-randomized (quasi-experimental)	Patients with depression	Depressed patients (*n =* 602) - Unipolar (*n =* 415) - Bipolar (*n =* 187)	Admission charts (3 years)	80%	Sunlight in patient rooms, either morning (max. 15,500 lux) or evening (max. 3000 lux)	ES	Recovery (length of stay)
Donald et al., 2015, Australia (82)	Qualitative (thematic analysis)	Patients (unspecified)	Patients (unspecified, *n =* 19)	Semi-structured interviews (*n =* 9); Focus groups (*n =* 10)	60%	Lack of privacy in glass treatment rooms, sterile (low stimulation) environment, lack of activity amenities	SS ES	Well-being (spatial confusion, boredom, feeling trapped, need for distraction)
Edwards and Hults, 1970, USA (83)	Mixed-methods, convergent design (quantitative non-randomized / descriptive analysis & qualitative descriptive/phenomenological)	Patients (unspecified) & staff	Staff (*n =* 26) Patients (*n =* 8)	No. patient interaction (time study); Questionnaires (patients & staff); In-depth interviews (patients); Clinical observations	67%	Closed vs. opened nursing station (removal of window glass)	S/A	Well-being (better verbal communication with staff, patient needs are better met, feeling less bothersome & threatening)
Haglund and von Essen, 2005, Sweden (84)	Qualitative (descriptive)	Patients (unspecified)	Patients (voluntary admitted; common diagnoses: mood d., schizophrenia, other psychotic d.s, anxiety, personality d.) (*n =* 20)	Semi-structured interviews	80%	Locked ward doors	EC S/A	Mental health (significant emotional distress and symptoms (state of panic, suicidal thoughts, nervousness, depression, fearfulness, anger), feeling dependent, decreased self-confidence, passiveness, feeling safe from the outside)
Kulkarni et al., 2014, Australia (85)	Quantitative non-randomized (cross-sectional)	Patients (female, unspecified)	Female patients (psychotic, mood d., post-partum psychosis/depr., anxiety d., eating d., personality d.) (*n =* 65) - intervention (*n =* 44) - control (*n =* 21) Staff (*n =* 20)	Safety incidents reports (6 months); Questionnaire (patients & staff)	80%	Female-only area	EC	Well-being (perceived safety and experience of care, satisfaction, comfort)
Lamanna et al., 2016, Canada (86)	Qualitative (interpretive theoretical framework)	Patients (unspecified) & staff	Patients (psychotic d., mood d., other) (*n =* 14) Staff (*n =* 10)	Semi-structured interviews	80%	Spatial confinement (if hospitalized involuntarily, secluded in their rooms, denied passes off the unit, or kept to scheduled passes)	EC S/A	Well-being (aggressive behavior fostered by feeling trapped, losing autonomy)
Muir-Cochrane et al., 2013, Australia (87)	Qualitative (phenomenological)	Patients held involuntarily, absconding experience/attempt (unspecified)	Former psychiatric inpatients, involuntarily admitted with absconding experience (*n =* 12)	Semi-structured interviews	80%	Crowding, noise, temperature discomfort, unpleasant aesthetics, calming surroundings (naturalness, color indoors), familiar/unfamiliar prison-like associations, mixed-gender settings; separate nurse station; facilities not promoting autonomy	SS ES EC S/A	Well-being (absconding behavior, comfort/discomfort, feelings of safety, healing association, boredom, lack of autonomy, psychological distance to staff)
Smith and Jones, 2014, UK (88)	Mixed-methods, sequential explanatory design (quantitative non-randomized / descriptive analysis; qualitative phenomenological)	Patients in PICU (acute disturbed phase, high risk to self/other safety, unspecified) & staff	PICU Patients (male, seclusion and sensory room experience, pathology not specified) (*n =* 7) Staff (*n =* 10)	No. seclusion incidences (3 months pre & post intervention); semi-structured interviews (13 months post intervention)	67%	Sensory room with equipment	ES	Mental health (perceived reduction in symptoms (staff and patients), calming and aiding de-escalation, relaxing and stress reducing, socialization, increased communication)
van Wijk et al., 2014, South Africa (89)	Qualitative (phenomenological)	Patients (unspecified)	Patients (not psychotic; *n =* 40; *n =* 20 each site)	Semi-structured interviews	100%	Crowding, noise, unhygienic conditions, seclusions rooms, mixed-pathology ward	SS ES EC S/A	Well-being (aggressive behavior, emotional distress)
Wood and Pistrang, 2004, UK (90)	Qualitative (phenomenological)	Patients (unspecified)	Patients (bipolar affective d., depression, schizophrenia, borderline p. d.) (*n =* 9) Staff (*n =* 7)	Semi-structured interviews (patient & staff)	100%	Mixed-gender wards, shared bedrooms, seclusion rooms	SS EC S/A	Well-being (feeling unsafe, vulnerable, threatened)
Connellan et al., 2015 (Australia) (47)	Qualitative (phenomenological)	Patients (unspecified) & staff	Patients and staff (*n =* unspecified)	Ethnographic observations based on 34 h of observation at morning and afternoon over a 10-week period	60%	Glass ration interior design (duty station and across ward), glass ratio interior design, glass	ES EC S/A	Well-being (actual and sense of safety, mesmerizing, distraction, confusion, lack of orientation)
Due et al., 2012 (Australia) (51)	Qualitative (phenomenological)	Patients (unspecified) & staff	Patients and staff (*n =* unspecified)	Ethnographic observations based on 34 h of observation at morning and afternoon over a 10-week period	60%	CCTV cameras as passive form of observation, availability/access to day-to-day facilities (food and drink), no access to personal belongings	EC	Well-being (aggressive behavior, being frightened and disturbed, comfortable/uncomfortable)
Riggs et al., 2013 (Australia) (50)	Qualitative (phenomenological)	Patients (unspecified) & staff	Patients and staff (*n =* unspecified)	Ethnographic observations based on 34 h of observation at morning and afternoon over a 10-week period	60%	Different designs of the nursing station (ratio of Glass, open/closed panel), CCTV; ledger; door on the side	S/A	Well-being (psychological distance between staff and patients/rehabilitative interaction, communication, feeling overly scrutinized)

*Study type categorization based on MMAT; Target population refers to study interest concerning patients [pathology], staff or both; Domain. SS, Social Stimulation; ES, Environmental Stimulation; EC, Environmental Control; S/A, Symbolism/Association. Outcome concerned mental health, well-being and treatment outcomes*.

#### Study Design and Methods of Analysis

The included 26 studies were heterogeneous in their study designs. The majority were qualitative (14 studies) and mixed-methods studies (four studies) using the following methods of analyses: Framework analysis = 1 (86), Grounded theory = 1 (76), Thematic analysis = 1 (82), Phenomenological analysis = 12 (49–51, 77–80, 83, 87–90), Content analysis = 3 (73, 75, 84). There were also quantitative non-randomized (eight studies) and mixed-method studies (four studies), primarily of cross-sectional design (Cross-sectional = 9; Quasi-Experimental = 2^6^; Descriptive = 2). Of these studies, two (22, 72) used correlational analyses (e.g., correlation, regression) to test the main hypotheses, four examined differences across conditions or groups (e.g., ANOVAs or *t*-tests) (72, 73, 75, 81, 84, 85, 91), one used multivariate analyses (70), one used multi-level modeling (74), and four reported descriptive statistics (70–75, 80, 81, 83–85, 88–91).

#### Sample Size

Included studies observed participants on an individual basis (20 studies) or on ward level (six studies). Of the individual level studies, the largest study included 1,227 participants, and the smallest study included six participants. Of the ward level studies, the largest study included 136 wards and the smallest study included two wards.

#### Sample Pathologies

Participant pathologies were not disclosed in 12 studies and not differentiated in nine studies. Five studies did differentiate by participant pathologies, however. These included: Females with history of sexual and/or physical abuse (one study), females who self-harm (one study), patients with depression (two studies), and patients with autistic spectrum condition (ASC) (one study).

#### Outcome Measures of Quantitative and Mixed-Methods Study

The quantitative component of the studies mostly used counts of inclusion, restraint, injection, or conflict incidences [seven studies; (22, 72–74, 80, 85, 88)]. Two studies used admission charts as a measure for length of stay (81, 91). Other studies used self-reports of violent ideation and behavior (70), Likert scale items on acceptability of door locking (71), and the number of patient interactions (83).

#### Outcomes Classified

With regard to observed outcomes, none of the studies specifically focused on mental health, well-being, or recovery outcomes, but reported on components of these concepts. Two studies (81, 91) reported on a proxy of recovery outcomes (shorter length of stay), six reported on aspects of mental health (e.g., worsening of symptoms or suicidal thoughts), and 18 reported on aspects of well-being (e.g., negative emotional states, comfort). Within the category of mental health, pathology specific studies reported on trauma-related significant emotional distress [states of panic, two studies; (75, 77)] and the worsening of symptoms [increased anxiety and self-harm, one study; (79)]. Mental health outcomes in pathology non-specific studies included perceived (by staff and/or patients) worsening or improvement of symptoms [one study; (88)], indicated recovery effects [one study; (71)], and significant emotional distress accompanied by suicidal thoughts [one study; (84)]. Well-being outcomes were found in non-pathology specific studies and included patient: Aggressiveness [seven studies; (22, 51, 70, 72, 76, 86, 89)]; sense of safety and security [seven studies; (49, 51, 78, 80, 87, 89, 90)]; absconding (escaping) behavior (one study; 76); range of negative affect [10 studies including anxiety, shame, vulnerability, boredom, discomfort, distress, spatial confusion; (49, 51, 73, 78, 80, 82, 85, 87, 89, 90)]; and aspects relating to rehabilitative or non-rehabilitative interaction with staff [six studies; (49, 50, 78, 83, 87, 90)]. Overall, none of the studies that provide pathology specific evidence reported on well-being outcomes, instead focusing on recovery or mental health. In contrast, none of the pathology-unspecific evidence reported on recovery outcomes.

### Quality Assessment

Quality appraisals for all studies can be found in the Supplementary Table 1. Overall, study quality was heterogeneous, with 46% being fair (10) or poor (2), and 54% of studies being rated excellent (5) or good (9). For the qualitative papers, the quality assessment scores ranged from 60 to 100% (average 80%). Most qualitative methods were justified, but others either chose a qualitative method not suitable for the research question (e.g., using ethnographic observer experiences of the environment to infer how the environment might affect patients in three studies) or recruited an inadequate sample (e.g., former patients whilst using a phenomenological approach). A significant number of studies were either weak in their analysis procedure or did not substantiate their interpretations with enough data. For the mixed-method papers, the quality assessment scores ranged from 47 to 80% (average 68%). Predominant weaknesses of the mixed-methods studies include an unsatisfactory integration of the different study component-methods (qualitative/quantitative) and non-adherence to the quality criteria of each component-method involved. For the quantitative papers, the quality assessment scores ranged from 40 to 80% (average 63%). Most studies outlined details of the research setting, but very few provided a clear justification of sample size. Furthermore, as some studies only provided a small amount of information about the sample characteristics (such as pathology), it was unclear whether the participants were representative of the target population. Most studies also failed to account for confounders in their design or analysis. A significant number of studies explored a range of environmental characteristics simultaneously and did not investigate discrete relationships between variables [e.g., investigating whether a change of the entire environmental context was associated with the number of compulsory restraint and injection incidences; (22)]. The most robust evidence was provided by two non-randomized, quasi-experimental studies on sunlight exposure and the length of hospitalization in patients with depression (81, 91). The quality assessments for the quantitative and mixed-methods studies were the lowest. The results from these studies therefore need to be interpreted cautiously.

Overall, the heterogeneity in study quality, the large amount of qualitative evidence (70% qualitative and mixed-methods), and the lack of robust quantitative designs, prevent the drawing of any conclusions about generalizable associations and directional cause-and-effect relationships between environmental characteristics and outcomes, and leaves the extracted evidence with significant risk of bias. As such, the review can only present indicative evidence of associations between environmental characteristics and health, well-being, and recovery outcomes in inpatients.

### Synthesized Findings of Pathology-Specific Evidence by Health Outcome

A summary of the pathology-specific results by health-related outcome and environmental characteristics, indicating study designs, can be found below in Table 3. Pathology-specific evidence is then presented by health outcomes (mental health and recovery outcomes, no evidence for well-being) and pathology. Evidence of components of mental health and recovery outcomes are highlighted in *italics*. Environmental characteristics and/or design elements are highlighted in **bold**.

**Table 3:** Findings of pathology-specific evidence on recovery, mental health and well-being.

**Dimension/Environmental characteristic**	**Health Outcomes**		
	**Recovery**	**Mental health**	**Well-being**
**Social stimulation**			
Crowding	^✗^	**Females who self-harm**: Trauma-related feelings of confusion and distress, panic, wishes to escape, sleep disruption [Lindgren et al., (77) - QLPH]	^✗^
Shared patient rooms incl. close observation area, beds in hallway	^✗^	**Females who self-harm**: Trauma-related feelings of confusion and distress, panic, wishes to escape, sleep disruption [Lindgren et al., (77) - QLPH]	^✗^
Mixed-gender ward	^✗^	**Females with history of abuse**: Trauma-related feelings of safety and control [Gallop et al., (75) - QLD]	^✗^
**Environmental stimulation**			
Noise	^✗^	**Females who self-harm**: Trauma-related feelings of confusion and distress, panic, wishes to escape, sleep disruption [Lindgren et al., (77) - QLPH];	^✗^
Multiple stimulation, stress inducing	^✗^	**ASC**: Anxiety and coping behavior [aggression, self-harm, social withdrawal; Maloret and Scott, (79) - QLPH]	^✗^
Sunlight in patient rooms	**Depression**: Shorter length of stay [Beauchemin and Hays, (91) - QTNRQX; Benedetti et al., (81) - QTNRQX]	^✗^	^✗^
**Control**			
Ward conditions affording little control	^✗^	**Females who self-harm**: Trauma-related feelings of confusion and distress, panic, wishes to escape, sleep disruption [Lindgren et al., (77) - QLPH]	^✗^
Facilities offering behavioral independence	^✗^	**Females with history of abuse**: Trauma-related feelings of safety and control [Gallop et al., (75) - QLD]	^✗^

*MMC, Mixed-methods convergent design; MMS, Mixed-methods; sequential explanatory design; QLD, Qualitative (descriptive); QLGT, Qualitative (grounded theory); QLPH, Qualitative (phenomenological); QLTF, Qualitative (interpretive theoretical framework); QLTA, Qualitative (thematic analysis); QTD, Quantitative (descriptive); QTNRCS, Quantitative non-randomized (cross-sectional); QTNRQX, Quantitative non-randomized (quasi-experimental);*

✗ *there were no findings associated with the environmental characteristics of recovery, mental health or well-being*.

Three studies offer pathology-specific analysis indicated environmental effects on mental health (75, 77, 79) and two on recovery outcomes (81, 91).

#### Mental Health

Females with history of sexual and/or physical abuse (75) and self-harm (77) reported *trauma-related emotional distress* as well as *trauma-related safety and control feelings* in relation to characteristics of the **social environment**. Those characteristics included lack of **privacy** lack of patient rooms, beds in hallway (77), **crowding** (77) and **mixed-gender** wards (75). Further, environmental stimulation stress was reported, which included **loud and frequent noises** (e.g., alert system) which was associated with *sleep disruption, panic, and the wish to escape*, particularly at night (77). **Environmental control** characteristics were prominent in both patient groups. The authors suggested that powerlessness and captivity can be central to the experience of women with a history of abuse and trauma (75). This included **poor ward conditions affording little environmental control** (no private rooms, no choice of spaces, locked doors) to protect themselves from unwanted stimuli or events (77), which was *considered not conducive to healing* (77). Patients wished for **soft lights** to improve night time visibility and increase comfort and control, as well as **alternative spaces** to use at night to increase the sense of control and alleviate nighttime concerns (75). Patients with autistic spectrum conditions (ASC) were reported (79) to be particularly affected by **environmental stimuli** on the ward (e.g., strong smells, bright lights, and air conditioning noise). This was associated with an *increase of symptoms in co-morbidities* (anxiety disorder) and *related coping strategies* (e.g., aggression, self-harm, social withdrawal).

#### Recovery Outcomes

Two studies addressed therapeutic enhancement by sensory stimulation; both relating to depression and **natural light** (81, 91). Beauchemin and Hays (91) compared the effects of sunlight and found that for hospitalized patients with depression, those in sunny rooms had a reduced *length of stay* compared with patients in dull rooms with decreased natural light. Benedetti et al. (81) found that morning exposure to sunlight in patient rooms resulted in *shorter stays* for bipolar depressed patients, compared with those exposed to evening natural light (an effect not observed in unipolar depressed patients).

In summary, there are very few studies (five) that indicated a relationship between environmental characteristics and recovery and mental health outcomes for specific psychopathologies. Only two studies provide fairly robust evidence on the isolated attributes of the physical environment and their impact on specific psychopathologies. These studies suggest that pathology specific sensitivity to environmental features [e.g., sensory vulnerability in patients with ASC, (92); trauma-related issues with control in patients with abuse] exist and should be considered. The review therefore finds a lack of pathology specific evidence.

### Synthesized Findings of Pathology-Unspecific Evidence on Mental Health

A summary of the pathology-unspecific results by health-related outcome and environmental characteristics, indicating study designs, can be found below in Table 4. In the subsequent text, evidence for the pathology-unspecific mental health outcomes is presented according to the four environmental domains of the previously presented framework (social stimuli, environmental stimuli, control, symbolism/associations). Please note, however, that there were no outcomes associated with social stimuli. Evidence of components of mental health are highlighted in *italics*. Environmental characteristics and/or design elements are highlighted in **bold**. Three studies indicated environmental effects on mental health (71, 84, 88).

**Table 4:** Findings of non-pathology-specific evidence on recovery, mental health and well-being.

**Dimension/Environmental characteristic**	**Health outcomes**		
	**Recovery**	**Mental health**	**Well-being**
**Social stimulation**			
Single patient room	^✗^	^✗^	Sanctuary place [(Muir-Cochrane et al., (87) - QLPH); Reduced aggressive behavior (Ulrich et al., (22) - QTNRCS)]
Shared patient rooms incl. close observation area, beds in hallway	^✗^	^✗^	Feeling unsafe, vulnerable [(Wood & Pistrang, (90) - QLPH; O'Brien and Cole, (80) – MMC_QTD&QLPH); Aggressive behavior (Ulrich et al., (22)- QTNRCS)]
Mixed-gender ward	^✗^	^✗^	Feeling unsafe, vulnerable [Wood & Pistrang, (90) - QLPH; O'Brien and Cole, (80) - MMC_QTD&QLPH; Muir-Cochrane et al., (87) - QLPH]
Too much privacy across the ward	^✗^	^✗^	Feeling unsafe [Muir-Cochrane et al., (87) - QLPH]
Lack of privacy in treatment rooms	^✗^	^✗^	Confusion [Donald et al., (82) - QLTA]
Crowding	^✗^	^✗^	Aggressive behavior [Brooks et al., (72) - QTNRCS; Ulrich et al., (22) - QTNRCS; Johnson and Delaney, (76) - QLGT; Van Wijk et al., (89) - QLPH]
Mixed-pathology ward	^✗^	^✗^	Aggressive behavior [Van Wijk et al., (89) - QLPH]
Seclusion room / forced isolation	^✗^	^✗^	Wish to socially connect, feelings of vulnerability, threat/fear, abandonment, anger, shame, sadness [Holmes et al., (78) - QLPH; Wood & Pistrang, (90) - QLPH]
**Environmental stimulation**			
Noise	^✗^	^✗^	Violent ideation [(Ben-Zeev et al., (70) - QTNRCS); Aggressive behavior (Van Wijk et al., (89) - QLPH), absconding behavior (Muir-Cochrane et al., (87) - QLPH)];
Environmental conditions	^✗^	^✗^	Absconding behavior [(Muir-Cochrane et al., (87) - QLPH); Aggressive behavior and emotional distress (Van Wijk et al., (89) - QLPH); Discomfort (O'Brien & Cole, (80) - MMC_QTD&QLPH)]
Glass	^✗^	^✗^	Feeling mesmerized, distracted, confused, lacking orientation [Connellan et al., (47) - QLPH]
Non-stimulating environment	^✗^	^✗^	Spatial confusion, boredom, need for distractions [Donald et al., (82) - QLTA]
Artwork complex /nature	^✗^	^✗^	Anxiety and agitation/calming [Nanda et al., (73) – MMC_QTNRCS&QLD]
Sensory room	^✗^	Perceived reduction in symptoms (staff/patients), calming [Smith & Jones, (88) -MMS_QTD/QLPH]	^✗^
Multiple stimuli, stress reducing	^✗^	^✗^	Reduction in aggressive behavior [(Ulrich et al., (22) - QTNRCS); Healing associations, reduction absconding (Mui-Cochrane et al., (87) - QLPH)]
**Control**			
Locked ward doors	^✗^	Emotional distress, recovery impacts [Bowers et al., (71) - QTD], emotional distress, anger, and self-harm [Haglund and von Essen, (84) - QLD]	Aggressive behavior [Bowers et al., (74) - QTNRCS]
Spatial confinement, mixed (incl. seclusion rooms)	^✗^	^✗^	Aggressive behavior [Lamanna et al., (86) - QLTF]; Vulnerability, threat/fear, abandonment, anger, shame, sadness [Holmes et al., (78) - QLPH; Wood & Pistrang, (90) - QLPH; Due et al., (51) – QLPH]; Vulnerability and threat; fear of physical abuse by staff [Van Wijk et al., (89) - QLPH]
CCTV vs. direct observation by staff	^✗^	^✗^	Comfortable/uncomfortable [Due et al., (51)- QLPH]
Visibility (ward design, location duty station, glass ratio interior)	^✗^	^✗^	Lower risk of aggressive behavior [Johnson and Delaney, (76) - QLGT]; Actual safety and sense of safety [Connellan et al., (49) - QLPH]
Mixed gender ward or area	^✗^	^✗^	Feeling unsafe, vulnerable [Wood & Pistrang, (90) - QLPH; O'Brien and Cole, (80) - MMC_QTD&QLPH; Muir-Cochrane et al., (87) - QLPH; Kulkarni et al., (85) - QTNRCS]
Facilities that offer freedom of choice	^✗^	^✗^	Absconding behavior, lack autonomy, boredom [Mui-Cochrane et al., (87) - QLPH]; Aggressive behavior [Ulrich et al., (22) - QTNRCS; Due et al., (51)]; Discomfort [O'Brien and Cole, (80) - MMC_QTD&QLPH; Due et al., (51)]
Ward conditions affording little control	^✗^	^✗^	Feeling traumatized and distressed [O'Brien and Cole - MMC_QTD&QLPH, (80) - MMC; Van Wijk et al., (89) - QLPH]
**Symbolism Home-like**			
Artwork complex/natural	^✗^	^✗^	Anxiety and agitation/calming [Nanda et al., (73) – MMC_TNRCS&QLD]
Familiarity	^✗^	^✗^	Absconding behavior, safety perceptions [Muir-Cochrane et al., (87) - QLPH]
**Symbolism Prison-like**			
Close observation area (fish-bowl design)	^✗^	^✗^	Safety perceptions [O'Brien and Cole, (80) - MMC_QTD&QLPH]
Locked ward doors	^✗^	Emotional distress, hinted recovery impacts [Bowers et al., (71) - QTD]; Emotional distress, aggressive behavior, self-harm [Haglund and von Essen, (84) - QLD]	^✗^
Spatial confinement (incl. seclusion room)	^✗^	^✗^	Aggressive behavior [Lamanna et al., (86) - QLTF]
Unfamiliarity/poor atmospheric qualities	^✗^	^✗^	Absconding behavior, safety perceptions, discomfort [Muir-Cochrane et al., (87) - QLPH]
**Psychological distance staff**			
Nurse station design	^✗^	^✗^	Communication, acceptance [Edwards and Hults, (83) -MMC_QTD&QLPH; Riggs et al., (50) – QLPH]; “them-us” relationship, overly scrutinized [Mui-Cochrane et al., (87) - QLPH; Riggs et al., (50); Connellan et al., (49) - QLPH], rehabilitative staff-patient interaction [Riggs et al., (50) – QLPH]
Locked ward doors	^✗^	Perception of “non-caring environment”, power-relationship [Haglund and von Essen, (84) - QLD]; Cold milieu hinting to hindered recovery [Bowers et al., (71) - QTD]	^✗^
Seclusion room	^✗^	^✗^	Feeling left alone and shamed [Holmes et al., (78) - QLPH; Wood & Pistrang, (90) - QLPH]; Vulnerability and threat; fear of physical abuse by staff [Van Wijk et al., (89) - QLPH]

*MMC, Mixed-methods, convergent design; MMS, Mixed-methods, sequential explanatory design; QLD, Qualitative (descriptive); QLGT, Qualitative (grounded theory); QLPH, Qualitative (phenomenological); QLTF, Qualitative (interpretive theoretical framework); QLTA, Qualitative (thematic analysis); QTD, Quantitative (descriptive); QTNRCS, Quantitative non-randomized (cross-sectional); QTNRQX, Quantitative non-randomized (quasi-experimental);*

✗ *there were no findings associated with the environmental characteristics of recovery, mental health or well-being*.

### Environmental Stimulation

Environmental stimulation was associated with mental health in one paper (88). This study found that **sensory rooms** with multiple stimulation options (light/image stimulation, music, reading, stress toys, place to socialize) was associated with a *perceived reduction in symptoms* (staff and patients) and *perceived drop-in seclusion rates* (quantitatively not verified) (88).

### Environmental Control

Environmental control was indirectly associated with mental health in two papers (71, 84). **Locked ward doors** and **spatial confinement/seclusion rooms** were associated with *emotional distress* (71), *worsening of symptoms and self-harm* (84) and *recovery impacts* (71).

### Symbolism/Associations

Environmental control was indirectly associated with mental health in two papers (71, 84), with **locked ward doors** creating prison-like associations and increasing the psychological distance to staff. This was associated with *significant emotional distress, aggressive behavior, and suicidal thoughts*. The authors suggested that locked doors created a “non-caring environment” (84) and cold milieu, which hindered recovery (e.g., hardening of staff feelings, greater authoritarianism, cold and controlling) (71), reflecting increased psychological distance between patients and staff.

In summary, there are very few pathology-unspecific studies (three) that indicate a relationship between environmental characteristics and mental health outcomes, and none on recovery outcomes. The studies provide no robust evidence on the effect of isolated attributes of the physical environment. The review therefore suggests a lack of evidence, particularly on mental health and recovery outcomes.

### Synthesized Findings of Pathology-Unspecific Evidence on Well-Being

Next, the pathology-unspecific evidence for well-being is presented along the four environmental domains. Evidence of components of mental health are highlighted in *italics*. Environmental characteristics and/or design elements are highlighted in **bold**. 18 studies indicated environmental effects on well-being (22, 49–51, 70, 72–74, 76, 78, 80, 82, 83, 85–87, 89, 90).

#### Social Stimulation

Social stimulation was addressed in nine papers (22, 72, 76, 78, 80, 82, 87, 89, 90) and refers to social overstimulation due to **lack of privacy** and **crowding**. It also includes an increased wish to **socially connect during forced isolation**. Associated outcomes were *feeling unsafe and vulnerable, aggressive behavior, rejection* and a *wish to connect with staff* .

**Lack of privacy** was reported in four studies as associated with *feelings of vulnerability, not being safe, and confusion*, and referred to several environmental characteristics, including **shared bedrooms** (22, 90), **mixed-gender wards** (80, 87, 90), observation bedrooms (80), and glass treatment rooms (82). Lack of privacy from shared bedrooms and mixed-gender wards was associated with patients feeling *unsafe* (87, 90) and *vulnerable* (80, 87, 90). Privacy in personal rooms was associated with a sense of *sanctuary* (87) and an aggression-reducing environment (22). Too much privacy throughout the ward was perceived as unsafe by some patients due to the opportunities for absconding and others entering without permission (87). Lack of privacy in treatment rooms (floor to ceiling glass on view to the ward) caused *confusion*, given the usual importance that privacy plays in consultations with health practitioners (82).

**Crowding** was reported in four studies (22, 72, 76, 89) to be associated with *aggressive behavior*. For instance, Brooks et al. (72) found in their comparative cross-sectional study of seven wards, that those at **overcapacity** (<9 meter^2^ of space per patient) reported more incidents of *aggressive behavior* (seclusion and constraint incidences). Johnson and Delaney (76) suggested that **public/common spaces should be of adequate size** (considering the number of patients and their psychopathological differing need for personal space) to enable roaming and emotional expression. Furthermore, van Wijk et al. (89) evaluated the accommodation of patients with **varying psychopathologies (mixed-pathology wards)** on the same crowded ward as problematic, due to the intensification of noise, conflict, and overreaching of rules.

**Forced isolation** through the use of **seclusion rooms** was associated with an *intensification of emotions* (78), a *range of negative emotions relating to rejection and abandonment*, and the *strong wish to socially connect to staff* (78, 90).

### Stress From Environmental Stimulation

Stress from environmental stimulation due to **noise**, a **combination of stimuli**, **abstract art**, **glass**, and **absence of stimulation** was addressed in seven studies (49, 70, 73, 80, 82, 87, 89). Associated outcomes were *violent ideation, aggressive behavior, absconding, anxiety, agitation, boredom*, and *confusion*.

**Noise** was reported in three studies. It was associated with *reduced ability to cope with stress* (89), *violent ideation* (70), *aggressive behavior* (89), and *absconding behaviors* (87), creating an overall unpleasant ward atmosphere (89). For instance, Ben-Zeev et al. (70) exclusively focused on noise, finding that **noisy ward conditions** were associated with increased odds of *violent ideation* in various types of high risk inpatients (schizophrenia, schizoaffective disorder, or bipolar disorder).

**Environmental conditions** were reported in three studies (80, 87, 89) and referred to **poor thermal comfort** (87), **unpleasant aesthetics** (87), **unhygienic conditions** (89), and poor levels of comfort [insufficient bathrooms and toilets, (80)], which was associated with *absconding behavior* (87), *aggression* (89) *and discomfort* (80). For instance, Van Wijk et al. (89) identified multiple aspects of the ward which contributed to an unpleasant atmosphere. Beyond the previously listed aspects of noise and crowding, they found that unhygienic conditions contributed to an environment that fostered *aggression*.

**Abstract art** was examined in one study. In Nanda et al.'s (73) study, nurse observations showed that patients reacted with *anxiety* and *agitation* to abstract art in the common room/patient lounge of an acute care psychiatric unit. Although reactions were not specified by psychopathology, nurses felt that patients who were already psychotic should not have exposure to disturbing (i.e., abstract) art. A comparison of PRN (pro re nata; as needed) medication administration for the reduction of anxiety and agitation, showed that medication use was significantly higher with exposure to abstract and representational art compared with realistic nature art.

**Glass** was investigated in one study, with its excessive use as a design or security element on the ward reported by Connellan et al. (49). Aside from creating visibility, it was found to be potentially distracting and overstimulating, by creating reflections and “duplications of overlapping and interpenetrating imagery” (p. 19). This resulted in an overstimulating, ambiguous space that was hard orientate in.

**Absence of stimulation** was reported in one study. Donald et al. (82) found that perceptions of a sterile environment and lack of amenities caused *feelings of boredom* in patients. The lack of amenities and ability to find *distractions* (“getting [mentally] away”) fostered feelings of *being institutionalized*. Additionally, the lack of environmental cues made patients feel *confused*.

### Therapeutic Enhancements by Sensory Stimulation

Three studies examined therapeutic enhancement by sensory stimulation through **natural art** (22, 73), and a combination of suggested **stress-reducing** attributes (22, 87), which were associated with reductions in *anxiety, agitation, aggression*, and *absconding*.

**Natural art** was directly observed or mentioned in two studies (22, 73). It was indirectly associated with a reduction in aggressive behavior [measured by injection rates; (22)], and directly associated with a reduction in *anxiety and agitation*. In Nanda et al. (73), nurses observed the *calming* effects of natural art in the common room/patient lounge on patients. The nurses believed that the nature-realistic artwork was a positive environmental element for their patients. PRN medication use for the reduction of *anxiety and agitation* was also significantly lower when exposed to realistic nature art compared with abstract and representational art.

A **combination of stress-reducing attributes** were mentioned in two studies (22, 87), which found that they were associated with *reduced aggression* (measured by injection rates) (22) and *healing associations*, and indirectly associated with a reduction in *absconding behavior* (87). For instance, Ulrich et al. (22) compared the proportion of patients requiring compulsory injections (an indicator of *aggressive behavior*) in two hospitals with different levels of stress-reducing environmental attributes. They found that injection rates were lower in the hospital with more stress-reducing attributes (single bedrooms, spatial communal areas with adjustable furniture, general low social density; noise reducing design, design for control in patient rooms; accessible garden, window with nature views, nature art, ample daylight; lines of sight from bedroom doors to communal areas). In an interview study by Muir-Cochrane et al. (87), patients associated healing with calming surroundings (natural surroundings outdoors and the use of color indoors).

### Environmental Control

Environmental control, including **spatial confinement**, **visibility**, **mixed-gender wards**, **facilities that offer choice**, and **lack of information control**, was examined in eleven studies (22, 49, 51, 74, 76, 78, 80, 85, 87, 89, 90). Outcomes associated with these variables included *aggressive behavior*, a *range of negative affect* and distress, feeling unsafe, *absconding, discomfort*, and feeling traumatized.

**Spatial confinement** and other security measures were found to lead to a loss of control, a sense of vulnerability, and aggression in six studies. Security measures included locked ward doors (74), seclusion room use, with or without security staff (51, 78, 89, 90), mixed forms of confinement [hospitalized involuntarily, secluded in their rooms, denied passes off the unit, or kept to scheduled passes; (86)], and CCTV (51). Locked ward doors were associated with *aggressive behavior* (74) and were considered less acceptable than constant observation but more acceptable than harsher containment [manual or chemical restraint or seclusion; (71)]. Loss of control during seclusion room use was associated with *emotional distress, shame, vulnerability*, and *fear* (78, 89, 90). Mixed forms of confinement were associated with *aggressive behavior*, fostered by the feeling of being trapped and a loss of autonomy (86). Related to this, Due et al. (51) described the *disturbing* and *anxiety* inducing effect of witnessing security personnel controlling non-compliant patient behavior; for example, a patient witnessing such a disturbing event was reported to lie down on the ground. Due et al. also found that patients preferred passive forms of observation (CCTV) rather than direct observation.

**Visibility** was mentioned in two studies. Johnson and Delaney (76) found that **visibility** through ward design and the strategic placing of nursing rooms (visibly present staff), the reduction of hidden spaces, and clear tangible boundaries of prohibited spaces (e.g., locking doors or boundaried rules regarding space use and access) were associated with a reduced risk of *violence*. They suggested that it reduces the chances of violent situations escalating, as well as giving an indirect sense of control (e.g., withdrawing behavior as exerting control). Connellan et al. (49) suggested that increased visibility through the extensive use of interior **glass** also promotes both *actual* and a perceived *sense of safety*.

**Mixed-gender wards** were related to a loss of control in four studies (78, 80, 90) and associated with *feelings of not being safe*. For instance, Kulkarni et al. (85) indicated that access to female only areas in mixed gender wards improved the sense of safety for female patients and reduced incidents which compromised safety.

A **lack of facilities that offer choice** and foster behavioral independence^7^ was associated with control in four studies (22, 51, 80, 87) and related to infrastructure for daily activities (e.g., food heating facilities, insufficient recreational spaces and activities). Associated outcomes included *absconding* (87), *aggressive behavior* (22), and *discomfort* (51, 80). For example, Muir-Cochrane et al. (87) explored the effects of a multitude of environment aspects on *absconding behavior*. They reported that a lack of freedom, fostered by denied autonomy, which was partly a result of spatial use and a lack of facilities to promote every day/“normal” activities, was one of the reasons given for patient *absconding*. The lack of freedom also led to boredom, which had negative effects on the therapeutic outcomes. This was evidenced by the quote: “there's nothing to do... you just smoke cigarettes” (p. 309).

**Lack of information control** was identified in two studies (80, 89). For example, being exposed to unclear hospital processes and limited information about its reasoning [cf. (4)], such as **witnessing seclusion** and **constraint incidences**, has been reported as *traumatizing* and *distressing* (80, 89). This was especially so if there was no possibility to withdraw due to the design specifications, such as in an eight-bedroom conversation area (80).

### Symbolism/Associations

Symbolism/associations have been addressed in ten studies, which examined either **home-like/prison associations** (73, 80, 86, 87) or **psychological distance to staff** (49, 50, 78, 83, 87, 89, 90). Associated outcomes were *aggressive behavior, absconding, discomfort*, and impacts on the staff-patient relationship (*communication, feelings of acceptance, shame, abandonment, vulnerability, and fear*).

**Home-like vs. prison** associations elicited by environmental characteristics were reported in four studies. Home-like associations were related to **natural art** (73) and **familiarity** (87), with the environment (e.g., knowing the staff, patients, and routines) perceived as *soothing* (73) and providing a *sense of safety* and *less reason to abscond* (87). Prison associations were related to **unfamiliarity**, **poor atmospheric qualities** (87), **spatial confinement** (69), and being housed in an **observation area** (80). Associated outcomes included feeling *unsafe* (80, 87), *discomfort* (87), *absconding* (87) and *aggressive behavior* (86). For instance, O'Brien and Cole (80) reported that patients associated **closed shared eight-bed observation areas** with a prison. This affected the sense of *privacy and security* of the patients, and researchers judged the design as threatening to their *physical and psychological safety*.

**Psychological distance** between staff and patients was reported in seven studies as accentuated by the environmental characteristics of **seclusion rooms** (78, 89, 90) and **nurse station design** [e.g., ratio of glass, open/closed panel, CCTV, ledger size, door on the side; (49, 50, 83, 87)]. Seclusion rooms underlined the stark power difference between staff and patients, which came with feelings of shame and abandonment (78, 90), and a great vulnerability and fear of abuse (89). The nurse station design impacted on both the staff-patient relationship (“them-us” relationship, feeling overly scrutinized) and communication (50, 83, 87). The use of glass in duty stations and across the ward was identified by Connellan et al., (49) as reinforcing the power-relationship, whereby staff are seen as visible but inaccessible. But it could also make patients feel more accepted (83) and overall, support the rehabilitative function of patient-staff interactions (50). For instance, Edwards and Hults (83) studied the effect of changing closed nurse station to an open design which resulted in reduced *psychological distance* between patients and staff. This improved patient-staff communication, staff were perceived to be more accessible, and staff did in fact spend less time in the nursing station and more time on the ward with the patients. Greater *accessibility* and better *communication* resulted in patients interrupting staff less and reducing the patient perception that staff were afraid of them. Patients *felt more accepted* for their special needs (less stereotyping by staff was found) and felt themselves less bothersome.

In summary, there are multiple (18) pathology-unspecific studies that indicate a relationship between environmental characteristics and well-being outcomes. However, none of them provide robust evidence. The review therefore demonstrates a lack of evidence and advanced study designs.

## Discussion

This systematic review synthesized qualitative, quantitative, and mixed-methods data from 26 studies, with the aim of examining the current state of empirical evidence on the influences of the physical and socio-physical environment on health-related outcomes (recovery, mental health, well-being) in mental healthcare inpatients by psychopathology. Despite the breadth of available research, the review revealed an underdeveloped evidence base, comprising studies of varying methodological quality, with few robust study designs. Although 19% of studies had an excellent method application, those were all purely qualitative papers; the two studies with the most robust designs (non-randomized quasi-experimental studies on the effect of day light on length of hospitalization in patients with depression) achieved quality ratings of fair and good. As such, the lack of rigorous experimental work does not allow any conclusions to be drawn about generalizable cause-and-effect relationships between environmental characteristics and patient outcomes; this makes it difficult to demarcate evidence-based practice [e.g., (93)]. Further, little evidence was retrieved on recovery outcomes and mental health, with most studies examining broad well-being outcomes such as aggression, sense of safety, and range of negative affect. Most also examined non-pathology specific (indicative) effects on mental health and well-being. Only five studies examined specific psychopathologies, potentially due to a lack of understanding of the specific architectural needs of diverse psychopathologies (31). This review shows that it is not possible to draw firm conclusions about the impact of specific physical or socio-environmental characteristics on psychiatric inpatients, especially not by pathology. This is surprising, given that previous literature reviews position the general evidence-base as reasonably strong [e.g., (94)].

The following sections discuss and reflect upon the research findings presented in this review, in combination with robust pathology-specific and unspecific evidence from other healthcare contexts and design recommendations. For clarity, the sections follow the structure of the analysis framework on person-environment fit (social stimulation, environmental stimulation, environmental control, symbolism/associations).

### Social Stimulation

The review found that privacy and crowding (the theme of forced isolation will be discussed in the following section on environmental control) were indicatively related to mental health outcomes in women who self-harm or have a history of abuse; both were related to well-being outcomes in the pathology-unspecific studies.

Unmet privacy needs were typically associated with patient rooms being shared (including in an eight-bed close observation area), rooms not being available (beds in hallway), and mixed-gender wards. Whereas pathology-unspecific studies suggested that poor person-environment fit, or privacy fit, is related to well-being outcomes (primarily to feeling unsafe and vulnerable), pathology-specific studies were able to provide more precise accounts of how the lack of control over social interaction (by the means of a single patient room) and mixed-gender wards affected their mental health. In the reviewed studies, women who self-harm and those with a history of abuse reported trauma-related feelings of confusion, distress, lack of safety, and sense of control. This was categorized as recovery-inhibiting by the studies' authors. This aligns with psychogeriatric studies, which highlight female inpatient vulnerability in mixed-gender psychiatric settings to threat, harassment and abuse by male patients (38). Single-gender rooms, areas, or wards is merited by some research (95, 96) and has been recommended in government policies [U.K. Department of Health policy to increase the provision of same sex wards for psychiatric patients; hospital services in NSW Australia; (38, 97)]. This could ease negative feelings experienced by some patients during their hospital stay, such as intrusion, embarrassment, or powerlessness, which results from crowding and a lack of privacy (38). However, others point out that, for example, in psychogeriatric research, the evidence is not yet adequate to mandate a *complete* gender segregation (38). Regarding private patient rooms, there is robust evidence from general healthcare settings highlighting the merit of private patient rooms. Benefits include; feelings of safety and reduced vulnerability, a sense of control and dignity, comfort and quality of life, and physiological health/recovery [cf. (98–100)]. It should be noted, however, that others have identified procedural complications related to single patient rooms [e.g., longer routes, making observations more difficult; (99)]. Robust psychopathology evidence excluded from this review (relating to dementia) identified a causal relationship between single person rooms and benefits, such as improved privacy and social interaction regulation. In this patient group, this was associated with reduced aggression, agitation and nervousness, and better sleep [e.g., (101)]. Given that psychiatric patients have been described as vulnerable to social (over) stimulation [e.g., (33)], require an environment providing dignity (7), and have particular needs due to their trauma-history, single patient rooms that allow privacy and regulated social interaction have been widely encouraged by researchers (18, 21, 29, 31, 39, 58, 101–103). Single patient rooms have been widely used in new inpatient units in, for example, Australia [cf. (38)]; despite being potentially unfavorable for historical or economic reasons (38). Historical concerns, including reduced treatment participation, increased social withdrawal, and lacking social patient interaction, seem to be unsubstantiated. In fact, it is large multiple-occupancy rooms that have been found to provoke patients' withdrawal (104). As such, some see a clear chance in single and non-dormitory bedrooms enhancing privacy and autonomy, and potentially promoting participation in treatment activities (38). However, we suggest caution toward “one-size-fits all approaches”, as some circumstances can justify room-in options [e.g., in cases in which a higher functioning roommate can provide aid to another in psychogeriatric settings; (105)].

The review indicated that social overstimulation from crowding was associated with actual or perceived social density, as well as mixed-pathology wards. In pathology-unspecific studies, this was primarily related to well-being, specifically aggressive behavior of patients against the self and/or fellow patients and/or staff members. Crowding was regarded as particularly problematic on mixed-pathology wards due to the intensification of noise, conflict, and overreaching of rules. The little pathology-specific evidence retrieved suggests that crowding contributes to a distressing environment, which hinders recovery in women who self-harm. Similarly, dementia research indicates that high-density settings are associated with aggression [e.g., (56, 101)]. Psychogeriatric research also indicates that segregating patients according to diagnosis results in significantly better care experiences for staff and patients [e.g., (96, 106)]. Some have concluded that providing separate wards or areas for patients with varying pathological needs can support treatment outcome, staff morale, and overall healthcare milieu [cf., (38)]. As such, some clinical practices already follow the model of providing pathology specific areas/wards [e.g., Clienia in Switzerland; cf. (107)]. The robustness of evidence on the relationship between social density/crowding and aggression-issues in general healthcare is primarily correlational evidence [e.g., (23, 47, 108, 109)], since experimental research is scarce and methodologically difficult to undertake [e.g., due to a necessary control of social withdrawal options; cf. (110)]. There is, however, robust evidence on physiological stress reactions (e.g., elevated blood pressure, heart rate, and skin conductance) and negative affect ratings in healthy populations [cf. (110)]. Since aggression is a continuous problem in psychiatric institutions (111–114), with some researchers identifying social density as a risk factor and arguing that aggression is prevalent in psychiatric patients (114–116), this aspect merits further exploration. Some practical advice given by researchers suggests that crowding issues might be easier to address in more modern facilities, where the design of larger indoor and outdoor spaces, or single bed units, for example, can be introduced more easily and as a priority in the design process (117, 118). Others add that structural design aspects should be considered in collaboration with certain management protocols, such as using separate areas for social interaction or limiting bed number availability per ward (38).

In summary, there is indicative evidence that socio-environmental fit regarding privacy and crowding is of pathology-specific (trauma and sensory resources related) and pathology unspecific relevance. General requirements indicated in this review concern single patient rooms, single-gender areas, optimal social capacity to prevent crowding, as well as pathology-specific ward sections. However, the lack of evidence suggests the need for further exploration of differing pathological needs (in relation to their trauma-history and/or sensory resources). This would enable evidence-based design recommendations to foster healing environments rather than hinder them.

### Demanding Environmental Stimulation

The review identified an indicative relationship between poor environment-fit (due to environmental overstimulation from features such as noise) and mental health outcomes in women who self-harm or patients with autistic spectrum condition (ASC). Poor stimulatory fit with regards to acoustical and visual overstimulation (e.g., noise, use of glass and abstract art), poor environmental conditions, as well as lack of stimulation, was associated with well-being outcomes in pathology-unspecific studies.

Noise overstimulation (defined as unwanted sound, typically characterized by intensity, frequency, periodicity, and duration) was unsurprisingly reported to induce anxiety and comorbid anxiety disorder, alongside associated coping behaviors (aggression, self-harm, social withdrawal) in ASC patients, due to their profound sensory vulnerabilities (92). Noise, particularly at night, induced self-harming patients into trauma-related states of panic and a wish to escape, as well as sleep disruption. Contrastingly, pathology-unspecific studies reported associations with aggressive and absconding behavior. Noise has gained attention in healthcare research (119–122) due to its effect on healing (e.g., immunosuppression, prolong wound healing, pain sensitivity, increased medication use) (123–126), especially when experienced as uncontrollable and unpredictable (irregular, sudden noises) (77, 110, 123). Some psychopathologies are, for neuropsychological reasons, clearly identifiable as sensorily vulnerable [e.g., dementia, neurodiverse conditions, schizophrenia; e.g., (7)]. For example, in dementia patients experiencing overstimulation by noise, negative behaviors and emotions such as violence and agitation (56, 101). Patient sensitivity to noise levels is reported as extremely high at times. Some have therefore argued that a high degree of acoustical control is required in spaces frequented by patients of psycho- and physiopathology institutions [e.g., (103, 117, 127)]. Others argue that fitting “helpful design features”, such as pleasant sounds or soundproofing, helps reduce sensory stress and offer positive environmental stimulation (38). Beyond the suggestion to provide separate wards for patients with specific sensory groups of pathology, other recommendations suggest providing a variety of areas and infrastructure to meet pathological-varying (sensory) needs [(38); e.g., compress rooms, multi-sensory rooms]. The latter solution seems most practical and feasible as it offers a certain flexibility in retrofitting existing ward designs/architecture.

Visual overstimulation, through abstract art and the use of glass in interior design, appeared to induce overload and confusion, resulting in increased anxiety and agitation in pathology unspecific studies. These results are consistent with the recommendations for patients with schizophrenia, which advise against the excessive use of glass to prevent unnecessary visual disturbances (doubling, distortions, and reflections), due to the categorization of impairment in these patients (7). However, researchers also point out that ideal levels of visual stimulation (e.g., bright colors and other visual stimulation cues) might vary between conditions, with depressive patients requiring more stimulation and activation whilst manic or cognitively impaired patients requiring less (31, 128). Regarding abstract art, relatively robust studies from general healthcare research also suggest that abstract art exposure results in increased negative emotions (e.g., worry, anger anxiety, depression) and psychophysiological stress responses (blood pressure and respiratory rate) (129–131) [cf. (132) for art-associated effects across various study designs].

In this review, recent studies reported on poor environmental conditions that result in thermal or hygienic discomfort. These arise across studies and countries (Australia and South Africa) where different public healthcare possibilities and standards exist. Environmental comfort (e.g., thermal comfort) and hygiene standards should be regarded as basic functional measures in healthcare, enabling recovery in psychopathological care (39). However, a recent review (38) on inquiries in Australia and the U.K. report a concerning lack of functional adequacy of public psychiatric facilities (e.g., U.K. Mental Health Act Commission, 2008: dirty, dangerous, overcrowded/lack of beds; Human Rights and Equal Opportunities Commission, 1993: poor design, overcrowded, lacking privacy and security; and inadequate toilet and bathing facilities). This indicates unsatisfactory standards across countries.

Lack of stimulation was found to be qualitatively associated with boredom, spatial confusion, and an increased desire for distraction. This echoes findings in healthy populations, with adequate stimulation levels afforded by the environment being required for various daily-life tasks and well-being [cf. (110)]. Lack of stimulation may also indicate a lack of spatial markers that support the readability, coherence, and comprehension of the space (e.g., orientation). This appears to be a common problem in institutional-looking healthcare settings, as indicated in research on patients with schizophrenia and dementia (7, 133, 134). However, severe forms of sensory deprivation have also been shown to be a serious threat to mental health and recovery outcomes. Sensory deprivation imposed by forced isolation has been found to aggravate psychotic symptoms [e.g., hallucinations, delusions, and paranoia; (135–137)].

In summary, there is indicative evidence that environmental fit (environmental over- or under-stimulation) is of pathology-specific (trauma and sensory resources related) and pathology unspecific relevance to mental health and well-being. General requirements indicated in this review include: the provision of conditions offering basic comfort on the ward (hygiene, thermal comfort); the prevention of unnecessary (137) arousal through acoustical (noise) or visual disturbances (excessive use of glass material, complex art); and the avoidance of the creation of a visually unstimulating environment that lacks, for example, spatial markers (7), and risks sensory deprivation. This collated evidence highlights the necessity of providing adequate sensory environments in psychiatric institutions (21, 31, 138). whilst being mindful of differing pathological needs in relation to trauma-history and sensory resources. However, an adequate stimulation level, or ideal person-environment fit, is likely to vary by pathology (e.g., the adequate level of stimulation being higher in patients with affect disorders than in patients being manic, having dementia, neurodiverse conditions, schizophrenia) which increases the complexity on a practical level. Whilst some suggest pathology-specific wards or sections, others suggest zones and amenities of varying sensory profiles (38). Considering the long-term exposure to stimuli in inpatient settings and the common lack of control over stimuli, health impacts could be significant. Moreover, considering the varying sensory needs by pathology, further research is required to develop pathology-specific evidence-based design recommendations.

### Therapeutic Environmental Stimulation

The review identified an indicative relationship between adequate environment-fit due to environmental stimulation (specifically natural light), and recovery outcomes (shorter stays) in patients with depression (varying effects by depression type). Other forms of adequate environmental stimulation, such as natural stimuli (accessible garden, nature window views, and nature art) and sensory rooms, were indicatively related to well-being and mental health outcomes in pathology-unspecific studies.

Natural and artificial light therapies have shown promising results in patients with various affect disorders [cf. (81)]. Pathology-specific evidence excluded from the review (dementia, forensics), also reports associations between light and sleep quality (139), symptomatic wandering behavior (56), seclusion incidences, and treatment outcomes (112). Considerably robust evidence can be found on the causal relationship between (natural/artificial) light and shorter hospital stays, amongst other impacts (e.g., mortality, medication, and sleep) (91, 139) in general healthcare research.

Regarding natural stimuli, robust, pathology-specific, experimental evidence excluded from this review also suggests that visual and acoustic natural stimuli can reduce violent incidences in dementia patients (140). General healthcare research demonstrates that natural stimuli exposures (view of natural scene with or without natural sounds, aquarium, virtual reality audiovisual nature, nature art) can decrease anxiety (130, 131, 141) and physiological distress (129, 142, 143), reduce the length of hospital stays (144) and affect pain levels, tolerance, and medication (144–147). In other contexts, access to outdoor gardens and other natural environments is found to bring positive effects, particularly through reduced stress and fatigue. Furthermore, large low windows can improve health outcomes and reduce delirium and paranoia (148). However, as discussed, stimulation levels can vary between patients and be too high for manic or cognitively impaired patients (38).

The review identified a study examining the benefits of a sensory room intervention which found mental health outcomes including a perceived reduction in symptoms (patients/staff) and other benefits reported by staff and patients (relieving stress, inducing sleep, reflecting, meditating, and belonging to a group). Robust evidence on patients with intellectual disabilities and/or autism spectrum disorder (ASD) in various healthcare contexts (assisted living, daycare facility, or hospital), demonstrates reductions in challenging behaviors, self-injuries, improved relaxation and treatment outcomes (149–153) when multisensory stimulation is available (e.g., multisensory therapy, snoezelen session, snoezelen room, relaxation therapy). The positive effects of calming environmental attributes, such as a decrease in pathological behavior, have also been identified in schizophrenic patients (154, 155).

In summary, environmental fit from adequate environmental stimulation is indicated to be of pathology-specific (natural light on depression types) and pathology unspecific relevance to recovery, mental health and well-being. The collated suggestive evidence corresponds to expert guidance for psychiatric environments, including recommendations for positive environmental stimuli such as natural light, nature exposure, and sensory rooms (24, 31, 39, 102, 138, 156), due to potential calming effects and reduction of restraint incidences. In fact, some position that the provision of a stimulating environment has become part of the therapeutic offer [(11, 12); e.g., on therapeutic use of sounds see (157–159)]. However, considering the varying sensory needs by pathology and the varying effects (as indicated in dementia research on environmental stimulation), as well as the lack of pathology-specific research, it would be premature to conclude this solely on the positive effects from this form of stimulation.

### Environmental Control

The review indicated associations between poor environmental fit, in relation to environmental control, and mental health outcomes in women who self-harm or have a history of abuse. Environmental control was indicatively related to mental health and well-being outcomes in pathology-unspecific studies.

For females both with and without a history of abuse or self-harm, ward conditions offering little control over social stimulation (e.g., crowding, male patients, beds in hallway) and environmental stimulation (e.g., night sounds, night flash light use by staff) as well as witnessing undesired events (e.g., others violent behavior), were considered unconducive to healing. This was indicated as particularly important for these patients, given that powerlessness can be central to experiences of women with a history of abuse and trauma, risking re-traumatisation (75). As such, Gallop et al. (75) points out that “the therapeutic milieu should be sensitive to the needs of women who have been abused … enabling them to attend to their need for safety and containment”. Acknowledgment, security, and containment are also considered essential features for treating trauma (160). Other research indicates that feelings of fear and trauma are related to time spent in wards with other disturbed or aggressive patients, highlighting the negative health impacts of being in an environment where undesired events are witnessed (38).

Pathology-unspecific evidence indicated that environmental conditions, which fostered a sense of control (e.g., visibility via ward design or glass material ratio, access to day-to-day facilities), were associated with well-being. Conditions which inhibited control (locked ward doors, mixed gender wards, various forms of spatial confinement) were primarily associated with negative behavior (aggression and absconding) and vulnerability. The suggestions posited by these studies correlate with recommendations from guidance documents and frameworks for reducing aggression and absconding rates, and improving recovery. These include options to withdraw from social and environmental stimuli (afforded, for example, by single person rooms), alongside enabling territoriality, increasing visibility, providing facilities which offer behavioral independence, promoting “normal” activities, and fostering competence (leisure rooms, Cafes, kitchens etc.) [(4, 19, 21, 25, 29, 39, 102), Klicken oder tippen Sie hier, um Text einzugeben. (156, 161)]. From a practical perspective, the provision of visibility to increase safety [actual and perceived; e.g., by strategic placement of nursing station, cf. (38)] whilst physically separating potentially risky patients is deemed important to consider, whilst maintaining a balance to provide patients with privacy and dignity (38).

An important theme in this review, and psychiatric care in general, is confinement. Mental health outcomes (e.g., recovery impacts and self-harm) have been associated with locked ward doors in two studies. Temporary confinement by imposing forced isolation, sometimes by security personnel, was also reported to have substantial well-being effects for those who witnessed the seclusion incidences (traumatizing, anxiety inducing), along with those who had to endure them. Experiencing forced isolation was associated with strong negative feelings (vulnerability, shame, abandonment) and the wish to connect to staff (see also association discussion). Forced isolation alongside chemical or physical constraint practices have been a common means to manage violent or self-harm behavior (19) and some researchers see therapeutic benefits (162). Others, however, are cautious and argue that the perceived loss of control and disempowerment can negatively affect mental health due to an associated loss of sense of self and a deterioration in the patient-staff relationship [cf. (7, 19, 87, 163)]. As for door locking practices in psychiatric facilities, this remains under debate, with unclear positions in the guidance (38). Some point to the risks of deteriorating the rehabilitative staff-patient relationship through these practices [e.g., (78), see association discussion]. Some specifically highlight the risk of inducing captivity trauma through locked wards, forced isolation, the use of restraint, and violence (164). For patients with a history of abuse, such confinements “can be experienced as a re-enactment of the abuse, perpetuating the sense of betrayal, insecurity, and powerlessness so central to the experience of women with a history of abuse” [(75), p.56]. Forced isolation and the resulting sensory deprivation has also been found to result in severe aggravation of psychotic symptoms (e.g., hallucinations, delusions, and paranoia; (135–137). Furthermore, research in other contexts, such as correctional facilities, points to severe psychological deterioration and mortality, especially through suicide, when inmates are under extreme and prolonged seclusion conditions (165). This indicates an ambivalence toward best practice for crisis situations (19) in psychiatric care, which requires urgent review given its health impacts.

In summary, environmental control is indicated to be of pathology-specific and pathology unspecific relevance to mental health and well-being. The collated suggestive evidence largely corresponds to expert guidance for psychiatric environments. These include urgent revision of confinement and constraint procedures, enabling territoriality and withdrawal, considering gender specific areas, creating visibility, providing facilities promoting “normal” activities and fostering competence. Design for control has become a widely recommended best practice in general health care for the reduction of unnecessary patient stress [e.g., (6, 22, 122, 125)]. However, providing psychiatric patients with a sense of control appears to be particularly important considering pathological-specific vulnerabilities (risk of re-traumatization), the risk of inducing captivity trauma, and deteriorating the rehabilitative staff-patient relationship across pathologies. Despite the need for control as a component of ensuring safety, it should be considered in a balance to maximize patient dignity and independence (38). Furthermore, as stays in inpatient facilities tend to be longer than those in physiopathological facilities, the impact is prolonged. As such, not fostering behavioral independence is a lost opportunity for treatment outcomes, given the potential to promote self-efficacy, autonomy, and the regaining of competency for the management of day to day life [e.g., (7, 12, 13, 19, 33)]. However, evidence elicited from this review is premature and further inquiries are needed for differentiated recommendations.

### Symbolism/Associations

The review indicated that home-like/prison associations and psychological distance with staff were associated with mental health and well-being outcomes in pathology-unspecific studies.

Home-like associations with the environment (e.g., nature art, familiar environmental features) were perceived as soothing and providing a sense of safety, whereas institutional, unfamiliar, or prison-like perceptions (e.g., mixed forms of spatial confinement, close observation area) were related to feeling unsafe, frightened, alien, uncomfortable and aggression inducing (well-being outcomes). Studies in the review indicated mental health outcomes (recovery impact and self-harm) from prison-like conditions and association as of locked ward doors. This corresponds to fairly robust studies in general healthcare environments, which reported positive effects of home-like features, such as feeling less confined (130, 166), more relaxed, secure, comfortable (167), and showing more positive affective appraisals and greater satisfaction (38, 168). A sense of familiarity (e.g., achieved by allowing a degree of ownership and personalization) has also been found to afford meaningfulness and coherence in the psychiatric hospital environment [e.g., (7, 12)]. As such, a home-like design is widely recommended as best practice (39, 156), with such recommendations also pointing toward the exterior environment [e.g., high fences have been associated with danger which can negatively impact patient relations to the hospital environment; (38)].

In the review, psychological distance between patients and staff was shown to be increased or reduced by environmental features relating to nurse station design, locked ward doors and seclusion room use. The removal of environmental barriers through opened nursing station design (e.g., removal of glass) was associated in with reduced psychological distance and improved rehabilitative staff-patient interaction. Seclusion room use was not only seen by patients as a punitive measure and modality for social control, but also resulted in a lack of nurse-patient contact. The authors suggested that this, in turn, explained the gravity of negative emotional experiences during seclusion, with coping strategies (regressing, acting out, compliance) motivated by the need to connect with staff (78). Overall, containment measures were found to underline the power relationship, fostering feelings of rejection and nurturing perceptions of a cold milieu hindering recovery.

In summary, associations and symbols are indicated to be of pathology unspecific relevance to mental health and well-being. Some collated suggestive evidence corresponds with expert guidance for psychiatric environments but leave ambivalent notions (see previous section on spatial confinements). As such, home-like designs and open nursing stations (with additional closed, locked spaces, and gathering spaces adjacent) have become a recommendation for psychiatric ward design to improve the social climate within and between patients and staff [e.g., (39, 156)]. Whilst recommendations for improving social climate appear unproblematic, recommendations for home-like design should be considered carefully. Not all pathologies might benefit from a home-like design that is visually complex and highly stimulating, as it may produce overstimulation (103), evident in dementia studies (57, 169). This suggests that achieving desired effects through well-meant interventions are complex, require careful consideration of the pathological profile of the patient, and thus, more research. The evidence also suggestively points to the importance of the relational aspects of nursing care, especially when applying this restrictive measure. Within the therapeutic milieu of the psychiatric setting, seclusion continues to be a widely used measure, despite being one of the most controversial management strategies (78). Researchers in the field state a requirement to better understand the nature of contact between staff and patients, establish true relationships, and move away from a “culture of control” toward a “culture of care” [e.g., (78)]. Thus, more research is required to better understand human-centered impacts of applying restrictive measures and find alternative solutions.

Overall, the review highlights a significant lack of robust evidence on isolated attributes of the physical environment and their impact on specific psychopathologies. This is a significant shortcoming of the field. Studies in this review point to indicative findings, which are partially echoed by more robust findings in other healthcare or non-healthcare contexts.

## Limitations

The study has limitations relating to the search strategy (search strings and database), handpicking strategy, strict inclusion/exclusion criteria as well as outcome domain categorization. The search string may have excluded relevant papers if they used different wording in the search terms, or mentioned the population in keywords or directly in the paper, rather than in the title or abstract. Specifying the mental healthcare facility as study object (S3) could also have limited the study retrieval. However, piloting without the S3 string elicited too many papers, which were logistically impossible to review. The combination of four search strings may also have constituted a reductionist approach. Furthermore, a database used in healthcare research (SCOPUS) was unavailable during the retrieval period, potentially limiting the retrieval range. The high number of handpicked sources might be indicative of retrieval limitations. Hand-picking was used to compliment the database searches and this technique relied on references found in items that were excluded due to format (e.g., systematic literature reviews or dissertations/theses). However, no additional handpicking was undertaken from journals or separate searches; apart from the exclusion of four titles *post-hoc* during the review process, as suggested by one reviewer. While the current review provides a good insight into the themes and qualities of the research, future reviews should consider utilizing a handpicking strategy that includes the bibliographies of key studies or frameworks in the field, and expert consultations for additional title retrieval. Overall, the difficulties of retrieving relevant literature signifies the lack of development in the field, which resulted in inconsistent vocabulary used to discuss the subjects (e.g., patients, clients, care receiver), different terminologies between disciplines, regions and times, and a lack of theoretical frameworks. These circumstances highlight the need to apply broader handpicking strategies, including expert consultation in future reviews. Another limitation of the current review is the strict inclusion/exclusion criteria, which resulted in the exclusion of a significant number of studies. Excluded studies were those that: focused on pathologies not listed in the Cochrane Framework of Common Mental Health Disorders (e.g., gerontopsychiatric studies); took place in forensic, rehabilitation, or ambulant/day-care settings; with mixed patient populations (adults and children); merged psychosocial (e.g., staff-patient relationship) and environmental characteristics in their analysis; were expert interviews and non-peer-reviewed (e.g., case studies). Lastly, the outcome domain categorization for mental health and well-being rested on the positioning of the evidence by the title authors. If authors were unclear about whether the evidence was symptomatic and pathology-related (mental health), the evidence was classified as relating to the well-being domain. As such, there is a risk that evidence might have been incorrectly classified. While this review may have limitations, its strengths can be found in the comprehensive database search, wide inclusion criteria of environmental attributes, and specific focus on the institutional setting (excluding psychogeriatric and forensic institutions), which address limitations in previous studies that lacked differentiation (170).

## Conclusions

This review examined the extent, nature, and quality of the current empirical evidence of the physical environment on mental health, well-being, and recovery outcomes in mental healthcare inpatients by psychopathology. Contrary to other reviews of the literature [e.g., (7, 94, 134)], our results indicate that the research, particularly regarding pathology, is generally not well developed, with studies only offering indicative effects, a consequence of non-robust study designs and methodological shortcomings. The majority do not focus on psychopathologies and their spatial requirements, nor specific environmental features, rather assessing many simultaneously. Given the potential of the physical hospital environment to support mental health and recovery outcomes, this review highlights the need for more methodologically sound research, capable of informing evidence-based guidelines for designing psychopathological-sensitive environments.

## Data Availability Statement

The original contributions presented in the study are included in the article/Supplementary Material, further inquiries can be directed to the corresponding author/s.

## Author Contributions

CW was principal investigator, who oversaw the process and work at different stages, and co-coordinated the research team. CW and VM contributed equally to search string design, screening, quality assessment, data extraction, analysis, and writing the manuscript. VM additionally performed the data retrieval. TW contributed to data extractions and co-writing the first draft of the manuscript. EM contributed to discussions of search strategy design, inclusion-exclusion criteria, analysis, and manuscript edits. EW contributed to quality assessment and data extraction (as external researcher) as well as editing the manuscript (as co-author). All authors contributed to the manuscript and approved the submitted version.

## Conflict of Interest

The authors declare that the research was conducted in the absence of any commercial or financial relationships that could be construed as a potential conflict of interest.

## Publisher's Note

All claims expressed in this article are solely those of the authors and do not necessarily represent those of their affiliated organizations, or those of the publisher, the editors and the reviewers. Any product that may be evaluated in this article, or claim that may be made by its manufacturer, is not guaranteed or endorsed by the publisher.

## Footnotes

^1^The exclusion of forensic institutions was informed by variability within forensic institutions across countries and between forensic and non-forensic institutions preventing comparability. Firstly, there are severe differences in forensic infrastructure and processes by country [e.g., (41)]. Secondly, due to access and infrastructure constraints imposed on the patients, a joined/undifferentiated analysis of non-forensic settings and forensic settings is not advisable.

^2^It is to note that Medline is indexed within PubMed and EBSCO (which includes CINAHL) has significant overlap with Ovid Index.

^3^Outcome domain categorization for mental health and well-being largely relied on the title-authors' positioning of the evidence. If authors' were unclear about whether the evidence is symptomatic and pathology-related, which would classify as mental health, the evidence was classified as well-being.

^4^Initially, the social stimulation dimension had another level that captures aspects that relate to increasing social interaction in space and incorporates the socio-fugal vs. socio-pedal space phenomena [cf. (4)]. However, no matching results were retrieved.

^5^Although defining mental health as the absence of mental illness has been considered too narrow when considering the concept on its own [e.g., (62)], this approach was considered valid for the present review, given our use of a broader well-being concept. However, well-being and mental health are considered associated constructs, not on a continuum [e.g., (63)].

^6^Arguably, both studies could also be classified as retrospective cohort studies [cf. (52)], although the studies' authors classified them as natural experiment.

^7^Several studies (51, 86, 89) report that loss of control over own belongings (for security reasons) fostered aggressive behavior. However, as these are procedural measures rather than environmental ones, those were not extracted for this review.

## References

[1] Canter D Canter S editors. Designing for Therapeutic Environments: A Review of Research. Chichester: Wiley (1979). p. 352.

[2] Gross R Yehuda S Architect MZ Zohar J. Healing environment in psychiatric hospital design general hospital. Psychiatry. (1998) 20:108–14. 10.1016/S0163-8343(98)00007-3 9582596

[3] Leventhal H Nerenz D Leventhal E. Illness and dehumanizing environments. In: Baum A, Singer JL, (eds) Advances in Environmental Psychology. (1982).

[4] Winkel GH Holahan CJ. The environmental psychology of the hospital: Is the cure worse than the illness? Prev Hum Serv. (1985) 4:11–33. 10.1080/10852358509511159 10274891

[5] Holahan CJ Saegert S. Behavioral and attitudinal effects of large-scale variation in the physical environment of psychiatric wards. J Abnorm Psychol. (1973) 82:454–62. 10.1037/h0035391 4770915

[6] Miedema E. Health promotive building design: Exploring perspectives on building design for health promotion in healthcare settings. Gothenburg: Chalmers University of Technology (2020).

[7] Golembiewski JA. Start making sense. Facilities. (2010) 28:100–17. 10.1108/02632771011023096

[8] Pelikan JM. The Application of Salutogenesis in Healthcare Settings. In: Mittelmark MB, Sagy S, Eriksson M, Bauer GF, Pelikan JM, Lindström B, et al., (eds.) The Handbook of Salutogenesis New York: Springer International Publishing. (2017). 10.1007/978-3-319-04600-6_25 28590659

[9] Lindern E Lymeus F Hartig T. The restorative environment: a complementary concept for salutogenesis studies. In: Mittelmark MB, Sagy S, Eriksson M, Bauer GF, Pelikan JM, Lindström B, et al., (eds). The Handbook of Salutogenesis. New York: Springer International Publishing. (2017). p. 181–95. 10.1007/978-3-319-04600-6_19 28590625

[10] Blumberg R Devlin AS. Design issues in hospitals. Environ Behav. (2006) 38:293–317. 10.1177/0013916505281575

[11] Dijkstra K. Understanding healing environments: effects of physical environmental stimuli on patients' health and well-being [dissertation]. Enschede: University of Twente. (2009).

[12] Devlin AS Arneill AB. Health care environments and patient outcomes. Environ Behav. (2003) 35:665–94. 10.1177/001391650325510214649024

[13] Marquardt G. Kriterienkatalog demenzfreundliche Architektur: Möglichkeiten der Unterstützung der räumlichen Orientierung in stationären Altenpflegeeinrichtungen Zugl. Dresden, Techn. Univ., Diss., 2006. Berlin: Logos-Verl.; 2007. p. 151. German.

[14] Brichetti K Mechsner F. Heilsame Architektur: Raumqualitäten erleben, verstehen und entwerfen. 1st ed. Bielefeld: transcript; (2019). p. 230. (Architekturen; vol. 48). German. 10.14361/9783839445037-fm

[15] Frandsen AK Mullins M Ryhl C Folmer MB Fich L. Helende arkitektur. Aalborg (2009). p. 29. Danish.

[16] Nickl-Weller C Nickl H. Healing Architecture. 1st ed. Salenstein: Braun (2013). p. 343. German.

[17] Nickl-Weller C editor. Healing Architecture. 2004-2017. 1st ed. Salenstein: Braun. (2017). p. 242. German.

[18] Platt LS Bosch SJ Kim D. Toward a Framework for Designing Person-Centered Mental Health Interiors for Veterans. J Inter Des. (2017) 42:27–48. 10.1111/joid.12095

[19] Chrysikou E. Architecture for Psychiatric Environments and Therapeutic Spaces. Amsterdam: IOS Press. (2014).

[20] Khanade K Rodriguez-Paras C Sasangohar F Lawley S. Investigating Architectural and Space Design Considerations for Post-Traumatic Stress Disorder (PTSD) Patients. Proc Human Factors Ergon Soc Ann Meet. (2018) 62:1722–6. 10.1177/1541931218621390

[21] Glasow N. Bauliche Suizidprävention in stationären psychatrischen Einrichtungen Zugl. Dresden, Techn. Univ., Diss., 2011. Berlin: Logos-Verl. (2011). p. 258. German.

[22] Ulrich RS Bogren L Gardiner SK Lundin S. Psychiatric ward design can reduce aggressive behavior. J Environ Psychol. (2018) 5:753–66. 10.1016/j.jenvp.2018.05.002

[23] Ulrich RS Bogren L Lundin S. Toward a design theory for reducing aggression in psychiatric facilities. In: ARCH12: Architecture / Research / Care / Health. (2012).

[24] DH Estates & Facilities. Achieving Excellence Design Evaluation Toolkit (AEDET). National Heath Service, UK. (2008).

[25] DH Estates & Facilities. A Staff and Patient Environment Calibration Toolkit (ASPECT). National Heath Service, UK. (2008).

[26] Dijkstra K Pieterse M Pruyn A. Physical environmental stimuli that turn healthcare facilities into healing environments through psychologically mediated effects: systematic review. J Adv Nurs. (2006) 56:166–81. 10.1111/j.1365-2648.2006.03990.x 17018065

[27] Gifford R Steg L Reser JP. Environmental Psychology. In: Martin PR, Cheung FM, Knowles MC, Kyrios M, Overmier JB, Prieto JM, (eds.). IAAP Handbook of Applied Psychology. Oxford, UK: Wiley-Blackwell. (2011). p. 440–70. 10.1002/9781444395150.ch18

[28] Zwarts A Coolen H. The meaning of preferences for residential environment features: a case study among appartment dwellers in the Netherlands. J Architec Plann Res. (2006) 23:200–15.

[29] EPH Psychiatrie. Evidenzbasiertes Planungshandbuch EPH Psychiatrie. 1st ed.: Universal Raum; (2012). German.

[30] Christenfeld R Wagner J Pastva G Acrish WP. How physical settings affect chronic mental patients. Psychiatr Q. (1989) 60:253–64. 10.1007/BF01064801 2641980

[31] Fricke OP Halswick D Längler A Martin DD. Healing architecture for sick kids. Z Kinder Jugendpsychiatr Psychother. (2019) 47:27–33. 10.1024/1422-4917/a000635 30560714

[32] Golembiewski JA. Salutogenic Architecture in Healthcare Settings. In: Mittelmark MB, Sagy S, Eriksson M, Bauer GF, Pelikan JM, Lindström B, et al., (eds.). The Handbook of Salutogenesis. New York: Springer International Publishing; 2017. p. 267–76. 28590652

[33] Nijman H Rector G. The crowded ward. PS. (1999) 50:1499–500. 10.1176/ps.50.11.1499-a8723190

[34] Matussek P. Psychopathologie II: Wahrnehmung, Halluzination und Wahn. In: Bally G, Brengelmann JC, Cornu F, Ey H, Eysenck HJ, Heimann H, et al. (eds.). Grundlagen und Methoden der Klinischen Psychiatrie. Berlin, Heidelberg: Springer; 1963. p. 23–76. German. 10.1007/978-3-662-00726-6_2

[35] Kunze M. Architekturpsychologische Untersuchungen zum Krankenhausbau [dissertation]. Weimar: Hoschschule für Architektur und Bauwesen (1994). German.

[36] Searles DH. The Non-human Environment. New York: International University Press. (1960).

[37] Chrysikou E Savvopoulou E. Implementing research and best practice for the development of mental health hubs in the community. London Bartlett Real Estate Institute. (2019). 10.1093/eurpub/ckaa165.1282

[38] Dobrohotoff JT Llewellyn-Jones RH. Psychogeriatric inpatient unit design: a literature review. Int Psychogeriatr. (2011) 23:174–89. 10.1017/S1041610210002097 21092352

[39] Karlin BE Zeiss RA. Environmental and therapeutic issues in psychiatric hospital design: toward best practices. PS. (2006) 57:1376–8. 10.1176/ps.2006.57.10.1376 17035554

[40] Page MJ McKenzie JE Bossuyt PM Boutron I Hoffmann TC Mulrow CD . The PRISMA 2020 statement: an updated guideline for reporting systematic reviews. BMJ. (2021) 29:525–35. 10.1136/bmj.n71 33781348 PMC8008539

[41] Biedermann A Champod C Willis S. Development of European standards for evaluative reporting in forensic science. Inte J Evid Proof. (2017) 21:14–29. 10.1177/1365712716674796

[42] Ulrich RS Berry LL Quan X Parish JT. A conceptual framework for the domain of evidence-based design. HERD. (2010) 4:95–114. 10.1177/193758671000400107 21162431

[43] Antonovsky A. Health, Stress, and Coping. 1st ed. San Francisco: Jossey-Bass Publ. (1979). p. 255.

[44] Vollmer T. Heilende Architektur - Wunsch oder Wirklichkeit. Ladenbrug: Bertha-Benz-Vorlesung (2018). p. 35. German.

[45] Ulrich RS Zimring C Zhu X DuBose J Seo H-B Choi Y-S . A review of the research literature on evidence-based healthcare design. HERD. (2008) 1:61–125. 10.1177/193758670800100306 21161908

[46] Brambilla A Rebecchi A Capolongo S. Evidence based hospital design. A literature review of the recent publications about the EBD impact of built environment on hospital occupants' and organizational outcomes. Ann Ig. (2019) 31:165–80. 10.7416/ai.2019.2269 30714614

[47] Connellan K Gaardboe M Riggs D Due C Reinschmidt A Mustillo L. Stressed spaces: mental health and architecture. HERD. (2013) 6:127–68. 10.1177/193758671300600408 24089185

[48] Elf M Anåker A Marcheschi E Sigurjónsson Á Ulrich RS. The built environment and its impact on health outcomes and experiences of patients, significant others and staff-A protocol for a systematic review. Nurs Open. (2020) 7:895–9. 10.1002/nop2.452 32257277 PMC7113518

[49] Connellan KA Riggs DW Due C. Light lies: how glass speaks. Commun Des Quart Rev. (2015) 3:15–24. 10.1145/2826972.2826974

[50] Riggs DW Due C Connellan KA. Duty stations and the regulation of space in mental health wards: a South Australian case study. Austral Commun Psychol. (2013) 25:78–93. Available online at: https://hdl.handle.net/2440/81585

[51] Due C Connellan K Riggs DW. Surveillance, security and violence in a mental health ward: an ethnographic case-study of a purpose-built unit in Australia. SS. (2013) 10:292–302. 10.24908/ss.v10i3/4.4276

[52] Hong QN Fàbregues S Bartlett G Boardman F Cargo M Dagenais P . The Mixed Methods Appraisal Tool (MMAT) version 2018 for information professionals and researchers. Educ Inf. (2018) 34:285–91. 10.3233/EFI-180221

[53] Popay J Roberts H Sowden A Petticrew M Arai L Rodgers M . Guidance on the conduct of narrative synthesis in systematic reviews: a product from the ESRC methods programme. Version. (2006) 1:b92. 10.13140/2.1.1018.4643

[54] Lawton MP. Environment and the need satisfaction of the aging. In Carstensen LL, Edelstein BB, editors. Handbook of Clinical Gerontology. Washington, DC: Pergamon Press (1987). p.33–40.

[55] Cvitkovich Y Wister A. Chapter 1 a comparison of four person-environment fit models applied to older adults. J Hous Elderly. (2001) 14:1–25. 10.1300/J081v14n01_01

[56] Algase DL Beattie ERA Antonakos C Beel-Bates CA Yao L. Wandering and the physical environment. Am J Alzheimers Dis Other Demen. (2010) 25:340–6. 10.1177/1533317510365342 20378834 PMC10845602

[57] Elmstahl S Annerstedt L Ahlund O Elmståhl S Ahlund O. How should a group living unit for demented elderly be designed to decrease psychiatric symptoms? Alzheimer Dis Assoc Disord. (1997) 11:47–52. 10.1097/00002093-199703000-00008 9071444

[58] Schultz R. Deconstructing the In-Patient Behavioral Health Facility: Finding the Balance Between Safety Therapeutics [master's thesis]. Clemson university. (2014). Available online at: https://tigerprints.clemson.edu/all_theses/2502/

[59] Lawton MP. Competence, environmental stress and the adaption of older people. In: Windley PG, Byerts TO, Ernst GF, (eds.) Theory development in environment and aging Washington: Gerontological Society. (1975).

[60] Oliveira-Maia AJ Mendonça C Pessoa MJ Camacho M Gago J. The mental health recovery measure can be used to assess aspects of both customer-based and service-based recovery in the context of severe mental illness. Front Psychol. (2016) 7:1679. 10.3389/fpsyg.2016.01679 27857698 PMC5093119

[61] Schrank B Slade M. Recovery in psychiatry. Psychiatr bull. (2007) 31:321–5. 10.1192/pb.bp.106.013425

[62] Keyes CLM. Mental illness and/or mental health? Investigating axioms of the complete state model of health. J Consult Clin Psychol. (2005) 73:539–48. 10.1037/0022-006X.73.3.539 15982151

[63] Lambert M Naber D Schacht A Wagner T Hundemer H-P Karow A . Rates and predictors of remission and recovery during 3 years in 392 never-treated patients with schizophrenia. Acta Psychiatr Scand. (2008) 118:220–9. 10.1111/j.1600-0447.2008.01213.x 18699954

[64] Ryff CD Singer B. The contours of positive human health. Psychol Inq. (1998) 9:1–28. 10.1207/s15327965pli0901_1

[65] Gatt JM Burton KLO Schofield PR Bryant RA Williams LM. The heritability of mental health and wellbeing defined using COMPAS-W, a new composite measure of wellbeing. Psychiatry Res. (2014) 219:204–13. 10.1016/j.psychres.2014.04.033 24863866

[66] Diener E Emmons RA Larsen RJ Griffin S. The satisfaction with life scale. J Pers Assess. (1985) 49:71–5. 10.1207/s15327752jpa4901_13 16367493

[67] Diener E Wirtz D Tov W Kim-Prieto C Choi D Oishi S . New well-being measures: short scales to assess flourishing and positive and negative feelings. Soc Indic Res. (2010) 97:143–56. 10.1007/s11205-009-9493-y

[68] Ryff CD. Happiness is everything, or is it? Explorations on the meaning of psychological well-being. J Person Soc Psychol. (1989) 57:1069–81. 10.1037/0022-3514.57.6.106926400043

[69] Ryff CD Keyes CLM. The structure of psychological well-being revisited. J Pers Soc Psychol. (1995) 69:719–27. 10.1037/0022-3514.69.4.719 7473027

[70] Ben-Zeev D Scherer EA Brian RM Mistler LA Campbell AT Wang R. Use of multimodal technology to identify digital correlates of violence among inpatients with serious mental illness: a pilot study. Psychiatric Services. (2017) 68:1088–92. 10.1176/appi.ps.201700077 28669285 PMC5891222

[71] Bowers L Haglund K Muir-Cochrane EC Nijman H Simpson A van der Merwe M. Locked doors: a survey of patients, staff and visitors. J Psychiatr Ment Health Nurs. (2010) 17:873–80. 10.1111/j.1365-2850.2010.01614.x 21078002

[72] Brooks K Mulaik JS Gilead M Daniels B. Patient overcrowding in psychiatric hospital units: effects on seclusion and restraint. Adm Policy Ment Health. (1994) 22:133–44. 10.1007/BF02106547

[73] Nanda U Eisen S Zadeh RS Owen D. Effect of visual art on patient anxiety and agitation in a mental health facility and implications for the business case. J Psychiatr Ment Health Nurs. (2011) 18:386–93. 10.1111/j.1365-2850.2010.01682.x 21539683

[74] Bowers L Allan T Simpson A Jones J van der Merwe M Jeffery D. Identifying key factors associated with aggression on acute inpatient psychiatric wards. Issues Ment Health Nurs. (2009) 30:260–71. 10.1080/01612840802710829 19363731

[75] Gallop R Engels S DiNunzio R Napravnik S. Abused Women's Concerns about Safety and the Therapeutic Environment during Psychiatric Hospitalization. Canad J Nurs Res. (1999) 31:53–70. 10696160

[76] Johnson ME Delaney KR. Keeping the unit safe: a grounded theory study. J Am Psychiatr Nurses Assoc. (2006) 12:13–21. 10.1177/1078390306286440

[77] Lindgren B-M Aminoff C Graneheim UH. Features of everyday life in psychiatric inpatient care for self-harming: an observational study of six women. Issues Ment Health Nurs. (2015) 36:82–8. 10.3109/01612840.2014.941077 25625707

[78] Holmes D Kennedy SL Perron A. The mentally ill and social exclusion: a critical examination of the use of seclusion from the patient's perspective. Issues Ment Health Nurs. (2004) 25:559–78. 10.1080/01612840490472101 15371143

[79] Maloret P Scott T. Don't ask me what's the matter, ask me what matters: Acute mental health facility experiences of people living with autism spectrum conditions. J Psychiatr Ment Health Nurs. (2018) 25:49–59. 10.1111/jpm.12438 29078024

[80] O'Brien L Cole R. Mental health nursing practice in acute psychiatric close-observation areas. Int J Ment Health Nurs. (2004) 13:89–99. 10.1111/j.1440-0979.2004.00324.x 15318903

[81] Francesco B Colombo C Barbini B Campori E Smeraldi E. Morning sunlight reduces length of hospitalization in bipolar depression. J Affect Diso. (2001) 62:221–3. 10.1016/S0165-0327(00)00149-X 11223110

[82] Donald F Duff C Lee S Kroschel J Kulkarni J. Consumer perspectives on the therapeutic value of a psychiatric environment. J Ment Health. (2015) 24:63–7. 10.3109/09638237.2014.954692 25915815

[83] Edwards J Hult MS. “Open” nursing stations on psychiatric wards. Perspect Psychiatr Care. (1970) 8:209–16. 10.1111/j.1744-6163.1970.tb01521.x 5203354

[84] Haglund K von Essen L. Locked entrance doors at psychiatric wards – Advantages and disadvantages according to voluntarily admitted patients. Nordic J Psychiat. (2005) 59:511–5. 10.1080/08039480500360781 16316906

[85] Kulkarni J Gavrilidis E Lee S van Rheenen TE Grigg J Hayes E . Establishing female-only areas in psychiatry wards to improve safety and quality of care for women. Australas Psychiatry. (2014) 22:551–6. 10.1177/1039856214556322 25358653

[86] Lamanna D Ninkovic D Vijayaratnam V Balderson K Spivak H Brook S . Aggression in psychiatric hospitalizations: a qualitative study of patient and provider perspectives. Aggression in psychiatric hospitalizations: a qualitative study of patient and provider perspectives. J Ment Health. (2016) 25:536–42. 10.1080/09638237.2016.1207222 27809615

[87] Muir-Cochrane EC Oster C Grotto J Gerace A Jones J. The inpatient psychiatric unit as both a safe and unsafe place: Implications for absconding. Int J. Mental Health Nurs. (2013) 22:304–12. 10.1111/j.1447-0349.2012.00873.x 23009358

[88] Smith S Jones J. Use of a sensory room on an intensive care unit. Use of a sensory room on an intensive care unit. J Psychosoc Nurs Mental Health Serv. (2014) 52:22–30. 10.3928/02793695-20131126-06 24305908

[89] van Wijk E Traut A Julie H. Environmental and nursing-staff factors contributing to aggressive and violent behavior of patients in mental health facilities. Curationis. (2014) 37:1122. 10.4102/curationis.v37i1.1122 25686162

[90] Wood D Pistrang N. A safe place? Service users' experiences of an acute mental health ward. J Commun Appl Soc Psychol. (2004) 14:304–12. 10.1002/casp.75525855820

[91] Beauchemin K Hays P. Sunny hospital rooms expedite recovery from severe and refractory depressions. J Affect Diso. (1996) 40:49–51. 10.1016/0165-0327(96)00040-7 8882914

[92] American Psychiatric Association. Diagnostic and statistical manual of mental disorders: DSM-5. 5th ed. Washington, DC: American Psychiatric Publishing (2013). p. 947. 10.1176/appi.books.9780890425596

[93] Briner RB Rousseau DM. Evidence-based I–O psychology: not there yet but now a little nearer? Ind organ psychol. (2011) 4:76–82. 10.1111/j.1754-9434.2010.01301.x

[94] Farahnak Majd N Golembiewski J Tarkashvand A. The psychiatric facility: how patients with schizophrenia respond to place. Design for Health. (2020) 4:384–406. 10.1080/24735132.2020.1846849

[95] Barlow F Wolson P. Safety and security: a survey of female psychiatric in-patients. Psychiatric bulletin. (1997) 21:270–2. 10.1192/pb.21.5.270

[96] Pollitt PA O'Connor DW. Are patients with severe depression traumatized by admission to an aged psychiatry ward? Int Psychogeriatr. (2007) 19:115–23. 10.1017/S1041610206003516 16928288

[97] Garling P. Final Report of the Special Commission of Inquiry. Acute Care Services in NSW Public Hospitals. (2008).

[98] Calkins M Cassella C. Exploring the cost and value of private versus shared bedrooms in nursing homes. Gerontologist. (2007) 47:169–83. 10.1093/geront/47.2.169 17440122

[99] Maben J Griffiths P Penfold C Simon M Anderson JE Robert G . One size fits all? Mixed methods evaluation of the impact of 100% single-room accommodation on staff and patient experience, safety and costs. BMJ Qual Saf. (2016) 25:241–56. 10.1136/bmjqs-2015-004265 26408568 PMC4819646

[100] Bilchik GS. A better place to heal. Health Forum J. (2002) 45:10–5. 12154637

[101] Morgan D Steward N. Multiple occupancy versus private rooms on dementia care units. Environ Behav. (1998) 30:487–503. 10.1177/001391659803000404

[102] Shepley MM Watson A Pitts F Garrity A Spelman E Kelkar J . Mental and behavioral health environments: critical considerations for facility design. Gen Hosp Psychiatry. (2016) 42:15–21. 10.1016/j.genhosppsych.2016.06.003 27638966

[103] Harris PB McBride G Ross C Curtis L. A Place to heal: environmental sources of satisfaction among hospital patients1. J Appl Social Pyschol. (2002) 32:1276–99. 10.1111/j.1559-1816.2002.tb01436.x

[104] Ittelson WH Proshansky HM Rivlin LG. Bedroom size and social interaction of the psychiatric ward. In: Wohlwill JF, Carson DH, editors. Environment and the social sciences: Perspectives and applications. Washington: American Psychological Association. (1972). p. 95–104. 10.1037/10045-009

[105] Folks DG Kinney FC. The medical psychiatry inpatient unit. In: Copeland JRM, Abou-Saleh MT, Blazer DG, editors. Principles and Practice of Geriatric Psychiatry: Wiley. (2002). p. 709–12. 10.1002/0470846410.ch129b25855820

[106] Craig J-S Patel J Lee-Jones C Hatton C. Psychiatric assessment wards for older adults: a qualitative evaluation of two ward models. Int J Geriat Psychiatry. (2000) 15:721–8 10.1002/1099-1166(200008)15:8<721::AID-GPS188>3.0.CO;2-K 10960884

[107] Clienia AG. Stationen [Internet]: Clienia AG. (2021). Available online at: https://www.clienia.ch/de/standorte/standorte-stationaer/zuerich/schloessli-oetwil-am-see/stationen. (accessed November 12, 2021)

[108] Cox VC Paulus PB McCain G. Prison crowding research: The relevance for prison housing standards and a general approach regarding crowding phenomena. Am Psychol. (1984) 39:1148–60. 10.1037/0003-066X.39.10.1148

[109] Paulus PB Cox VC McCain G. Prisons Crowding: A Psychological Perspective. New York, NY: Springer New York. (1988). 10.1007/978-1-4612-3812-6

[110] Evans GW Cohen S. Environmental Stress. In: Stockols D, Altman I, editors. Handbook of Environmental Psychology: Wiley. (1987). p. 571–610.

[111] Heinze C Gaatz S Dassen T. Aggression in psychiatrischen Kliniken. Psych Pflege Heute. (2005) 11:149–53. 10.1055/s-2005-85793126606158

[112] Olver J Love M Daniel J Norman T Nicholls D. The impact of a changed environment on arousal levels of patients in a secure extended rehabilitation facility. Australas Psychiatry. (2009) 17:207–11. 10.1080/10398560902839473 19404817

[113] Finzel M Schmidmeier R Fric M Widauer M Laux G. Aggressionen psychiatrischer Patienten - Erste Ergebnisse einer standardisierten Dokumentation des BZK Gabersee [Violence by Psychiatric In-Patients of the BZK Gabersee - First Results of a Standardized Documentation]. Psychiatr Prax. (2003) 30:196–9. 10.1055/s-2003-39767 13130374

[114] Ketelsen R Zechert C Driessen M Schulz M. Characteristics of aggression in a German psychiatric hospital and predictors of patients at risk. J Psychiatr Ment Health Nurs. (2007) 14:92–9. 10.1111/j.1365-2850.2007.01049.x 17244011

[115] Schanda H Taylor P. Aggressives Verhalten psychisch Kranker im stationären Bereich: Häufigkeit, Risikofaktoren, Prävention [Inpatient violence: frequency, risk factors, preventive strategies]. Fortschr Neurol Psychiatr. (2001) 69:443–52. 10.1055/s-2001-17560 11602920

[116] Cheung P Schweitzer I. Correlates of aggressive behaviour in schizophrenia: an overview. Aust N Z J Psychiatry. (1998) 32:400–9. 10.3109/00048679809065534 9672731

[117] Bergsland K. Space in mind. J Healthc Des Develop. (2002) 3:318–24.

[118] Kumar S Ng B. Crowding and violence on psychiatric wards: explanatory models. Can J Psychiatry. (2001) 46:433–7. 10.1177/070674370104600509 11441783

[119] Aaron JN Carlisle CC Carskadon MA Meyer TJ Hill NS Millman RP. Environmental noise as a cause of sleep disruption in an intermediate respiratory care unit. Sleep. (1996) 19:707–10. 10.1093/sleep/19.9.707 9122557

[120] Scalise D. Patient care. Shhh, quiet please! Hosp Health Netw. (2004) 78:16–7. 15192873

[121] Hilton BA. Noise in acute patient care areas. Res Nurs Health. (1985) 8:283–91. 10.1002/nur.4770080311 3852363

[122] Steptoe A Appels A (eds.). Stress, personal control and health. Chicester: Wiley. (1989). vol. 7.

[123] Topf M Davis JE. Critical care unit noise and rapid eye movement (REM) sleep. Heart Lung. (1993) 22:252–8. 8491660

[124] Biley FC. Effects of noise in hospitals. Br J Nurs. (1994) 3:110–3. 10.12968/bjon.1994.3.3.110 8148625

[125] Nelson Montague K Sharrow R. Healing Environments: Creating a Nurturung and Healthy environment. In: Frampton SB, Chalmer PA, Planetree, (eds.) Putting Patients First: Best Practices in Patient-Centered Care. San Francisco Calif: Jossey-Bass. (2009).

[126] Hagerman I Rasmanis G Blomkvist V Ulrich RS Eriksen CA Theorell T. Influence of intensive coronary care acousticson the quality of care and physiological state of patients. Int J Cardiol. (2005) 98:267–70. 10.1016/j.ijcard.2003.11.006 15686777

[127] Lawton MP Fulcomer M Kleban MH. Architecture for the mentally impaired elderly. Environ Behav. (1984) 16:730–57. 10.1177/0013916584166004

[128] Grant LA. Beyond the dichotomy. Res Aging. (1998) 20:569–92. 10.1177/0164027598205002

[129] Eisen S. Effects of Art in Pediatric Healthcare. [dissertation] Texas: Texas A&M University. (2006).

[130] McCabe C Roche D Hegarty F McCann S. 'Open Window': a randomized trial of the effect of new media art using a virtual window on quality of life in patients' experiencing stem cell transplantation. Psychooncology. (2013) 22:330–7. 10.1002/pon.2093 22147646

[131] Ulrich RS Lundéen O Eltinge JL. Effects of exposure to nature and abstract pictures on patients recovering from heart surgery. Psychophysiology. (1993) 30:7.

[132] Ulrich RS. Effects of viewing art on health outcomes. In: Frampton SB, Charmel P, editors. Putting Patients First. San Francisco: Jossey-Bass. (2009). p. 129–49.

[133] Hanley IG. The use of signposts and active training to modify ward disorientation in elderly patients. J Behav Ther Exp Psychiatry. (1981) 12:241–7. 10.1016/0005-7916(81)90053-7 7320213

[134] Golembiewski J. There's something in my head (but it's not me): The complex relationship between the built environment schizophrenia - from aetiology to recovery. Sydney. Australia: Sydney University. (2012). Available online at: https://www.academia.edu/2646010/There_s_something_in_my_head_but_it_s_not_me_The_complex_relationship_between_the_built_environment_and_schizophrenia

[135] Richardson BK. Psychiatric inpatients' perceptions of the seclusion-room experience. Nurs Res. (1987) 36:234–7. 10.1097/00006199-198707000-00012 3648697

[136] Wadeson H Carpenter WT. Impact of the seclusion room experience. J Nerv Ment Dis. (1976) 163:318–28. 10.1097/00005053-197611000-00004 789819

[137] Baradell JG. Humanistic care of the patient in seclusion. J Psychosoc Nurs Ment Health Serv. (1985) 23:8–9. 10.3928/0279-3695-19850201-04 3845118

[138] Wilson C Rouse L Rae S Kar Ray M. Mental health inpatients' and staff members' suggestions for reducing physical restraint: a qualitative study. J Psychiatr Ment Health Nurs. (2018) 25:188–200. 10.1111/jpm.12453 29323442

[139] Campbell SS Kripke DF Gillin J Hrubovcak JC. Exposure to light in healthy elderly subjects and alzheimer's patients. Physiol Behav. (1988) 42:141–4. 10.1016/0031-9384(88)90289-2 3368532

[140] Whall AL Black ME Groh CJ Yankou DJ Kupferschmid BJ Foster NL. The effect of natural environments upon agitation and aggression in late stage dementia patients. Am J Alzheimer's Dis. (1997) 12:216–20. 10.1177/153331759701200506

[141] Katcher A Segal H Beck A. Comparison of contemplation and hypnosis for the reduction of anxiety and discomfort during dental surgery. Am J Clin Hypn. (1984) 27:14–21. 10.1080/00029157.1984.10402583 6391137

[142] Ulrich RS Simons RF Miles MA. Effects of environmental simulations and television on blood donor stress. J Architect Plann Res. (2003) 20:38–47. Available online at: http://www.jstor.org/stable/43030641

[143] Schneider SM Prince-Paul M Allen MJ Silverman P Talaba D. Virtual reality as a distraction intervention for women receiving chemotherapy. Oncol Nurs Forum. (2004) 31:81–8. 10.1188/04.ONF.81-88 14722591

[144] Ulrich RS. View through a window may influence recovery from surgery. Science. (1984) 224:420–1. 10.1126/science.6143402 6143402

[145] Diette GB Lechtzin N Haponik E Devrotes A Rubin HR. Distraction therapy with nature sights and sounds reduces pain during flexible bronchoscopy: a complementary approach to routine analgesia. Chest. (2003) 123:941–8. 10.1378/chest.123.3.941 12628899

[146] Lee DWH Chan ACW Wong SKH Fung TMK Li ACN Chan SKC Mui LM . Can visual distraction decrease the dose of patient-controlled sedation required during colonoscopy? A prospective randomized controlled trial. Endoscopy. (2004) 36:197–201. 10.1055/s-2004-814247 14986215

[147] Tse MMY Ng JKF Chung JWY Wong TKS. The effect of visual stimuli on pain threshold and tolerance. J Clin Nurs. (2002) 11:462–9. 10.1046/j.1365-2702.2002.00608.x 12100642

[148] Ulrich RS. Effects of gardens on health outcomes: theory and research. In: Cooper-Marcus C, Barnes M, (eds.) Healing Gardens: Therapeutic Benefits and Design Recommendations New York: John Wiley. (1999).

[149] Lindsay WR Pitcaithly D Geelen N Buntin L Broxholme S Ashby M . comparison of the effects of four therapy procedures on concentration and responsiveness in people with profound learning disabilities. J Intellect Disabil Res. (1997) 41:201–7. 10.1111/j.1365-2788.1997.tb00698.x 9219068

[150] Shapiro M Parush S Green M Roth D. The efficacy of the “snoezelen” in the management of children with mental retardation who exhibit maladaptive behaviours. Br J Develop Disab. (1997) 43:140–55. 10.1179/bjdd.1997.01415017085

[151] Cuvo AJ May ME Post TM. Effects of living room, Snoezelen room, and outdoor activities on stereotypic behavior and engagement by adults with profound mental retardation. Res Dev Disabil. (2001) 22:183–204. 10.1016/S0891-4222(01)00067-1 11380058

[152] Kaplan H Clopton M Kaplan M Messbauer L McPherson K. Snoezelen multi-sensory environments: task engagement and generalization. Res Dev Disabil. (2006) 27:443–55. 10.1016/j.ridd.2005.05.007 16122905

[153] Novakovic N Milovancevic MP Dejanovic SD Aleksic B. Effects of Snoezelen-Multisensory environment on CARS scale in adolescents and adults with autism spectrum disorder. Res Dev Disabil. (2019) 8:951–8. 10.1016/j.ridd.2019.03.007 30933867

[154] Gabb BS Speicher K Lodl K. Environmental design for individuals with schizophrenia: an assessment tool. sgrjarc. (1992) 23:35–40. 10.1891/0047-2220.23.2.35

[155] Higgs WJ. Effects of gross environmental change upon behavior of schizophrenics: a cautionary note. J Abnorm Psychol. (1970) 76:421–2. 10.1037/h0020134 5490708

[156] Hunt JM Sine DM. Design Guide for the Built Environment of Behavioral Health Facilities. National Association of Psychiatric Health Systems. (2017).

[157] Williamson JW. The effects of ocean sounds on sleep after coronary artery bypass graft surgery. Am J Crit Care. (1992) 1:91–7. 10.4037/ajcc1992.1.1.91 1307884

[158] Ferguson E Singh AP Cunningham-Snell N. Stress and blood donation: effects of music and previous donation experience. Br J Psychol. (1997) 88:277–94. 10.1111/j.2044-8295.1997.tb02635.x 9183841

[159] Thorgaard B Henriksen BB Pedersbaek G Thomsen I. Specially selected music in the cardiac laboratory-an important tool for improvement of the wellbeing of patients. Eur J Cardiovasc Nurs. (2004) 3:21–6. 10.1016/j.ejcnurse.2003.10.001 15053885

[160] Kirby JS Chu JA Dill DL. Correlates of dissociative symptomatology in patients with physical and sexual abuse histories. Compr Psychiatry. (1993) 34:258–63. 10.1016/0010-440X(93)90008-R 8348805

[161] Welter R. Architektur, gewalt und aggression in kliniken. Syst Familie. (1997) 10:88–91. 10.1007/s004910050009

[162] Malkin J. Hospital interior architecture: Creating healing environments for special patient populations. New York, NY: VanNostrand Reinhold. (1992). p. 478.

[163] Kuosmanen L Makkonen P Lehtila H Salminen H. Seclusion experienced by mental health professionals. J Psychiatr Ment Health Nurs. (2015) 22:333–6. 10.1111/jpm.12224 26014830

[164] Cohen LJ. Psychiatric hospitalization as an experience of trauma. Arch Psychiatr Nurs. (1994) 8:78–81. 10.1016/0883-9417(94)90037-X 8042870

[165] Luigi M Dellazizzo L Giguère C-É Goulet M-H Dumais A. Shedding light on “the Hole”: a systematic review and meta-analysis on adverse psychological effects and mortality following solitary confinement in correctional settings. Front Psychiatry. (2020) 11:840. 10.3389/fpsyt.2020.00840 32973582 PMC7468496

[166] Olsen R. The effect of the hospital environment: patient reaction to traditional versus progressive care settings. J Archit Plann Res. (1984) 1:121–36.

[167] Ingham B Spencer C. Do comfortable chairs and soft lights in the waiting area really help reduce anxiety and improve the practice's image? Health Psychol Update. (1997) 2:817–20.

[168] Leather P Beale D Santos A Watts J Lee L. Outcomes of environmental appraisal of different hospital waiting areas. Environ Behav. (2003) 35:842–69. 10.1177/0013916503254777

[169] Vaaler AE Morken G Linaker OM. Effects of different interior decorations in the seclusion area of a psychiatric acute ward. Nord J Psychiatry. (2005) 59:19–24. 10.1080/08039480510018887 16195094

[170] Sheehan B Burton E Wood S Stride C Henderson E Wearn E. Evaluating the built environment in inpatient psychiatric wards. Psychiatr Serv. (2013) 64:789–95. 10.1176/appi.ps.201200208 23632426

