# Supplementary material for: Patients' Health & Well-Being in Inpatient Mental Health-Care Facilities: A Systematic Review
Source: Front Psychiatry. 2022 Jan 3;12:758039. doi: 10.3389/fpsyt.2021.758039 (PMC8761847; doi:10.3389/fpsyt.2021.758039)
Supplement: Supplementary file 1 [file Table_1.DOCX]

Table 1 | Quality Assessment

| MMAT for mixed method | | Wood & Pistrang, 2004, UK (977) | Van Wijk et al., 2014, South Africa (857) | Maloret & Scott, 2018, United Kingdom (367) | | Lamanna et al., 2016, Canada (726) | | Holmes et al., 2004, Canada (267) | Donald et al., 2015, Australia (525) | | Beauchemin & Hays, 1996, Canada (51) | Benedetti et al., 2001, Unknown location (209) | Kulkarni et al., 2014, Australia (713) | | Smith & Jones, 2014, UK (546) | Gallop et al., 1996, Canada (719) | Ben-Zeev et al., 2017, USA (59) | Brooks et al., 1994, USA (93) | Muir-Cochrane et al., 2013, Australia (406) | | Lindgren et al., 2015, Sweden (838) | | Bowers et al., 2009, England (859) | Johnson & Delaney, 2006, USA (881) | Bowers et al., 2010, UK (83) | O'Brien & Cole, 2004, Australia (354) | | Ulrich et al., 2018, Sweden (627) | Nanda et al., 2011, USA (809) | Haglund & von Essen, 2005, Sweden (259) | Edwards & Hults, 1970, USA (280) | | | Connellan et al., 2015, Australia | | Due et al., 2012, Australia | Riggs et al., 2013, Australia | |
| --- | --- | --- | --- | --- | --- | --- | --- | --- | --- | --- | --- | --- | --- | --- | --- | --- | --- | --- | --- | --- | --- | --- | --- | --- | --- | --- | --- | --- | --- | --- | --- | --- | --- | --- | --- | --- | --- | --- |
| Overall Score | | 100.0% | 100.0% | 80.0% | | 80.0% | | 100.0% | 60.0% | | 60.0% | 80.0% | 80.0% | | 66.7% | 80.0% | 60.0% | 60.0% | 80.0% | | 100.0% | | 40.0% | 100.0% | 60.0% | 80.0% | | 80.0% | 46.7% | 80.0% | 66.7% | | | 60% | | 60% | 60% | |
| Qualitative (ethnography, phenomenology, narrative research, grounded theory, case study, qualitative descriptive study) | 1.1 Is the qualitative approach appropriate to answer the research question? | Yes | Yes | | Yes | | Yes | Yes | | Yes |  |  | |  | Can't tell | Yes |  |  | | Yes | | Yes |  | Yes |  | | Yes |  | Yes | Yes | | Yes | Yes | | Yes | | | Yes |
|  | 1.2 Are the qualitative data collection methods adequate to address the research question? | Yes | Yes | | No | | Can't tell | Yes | | Yes |  |  | |  | Yes | Yes |  |  | | No | | Yes |  | Yes |  | | Yes |  | Yes | Yes | | Yes | No | | No | | | No |
|  | 1.3 Are the findings adequately derived from the data? | Yes | Yes | | Yes | | Yes | Yes | | Can't tell |  |  | |  | Yes | No |  |  | | Yes | | Yes |  | Yes |  | | Can't tell |  | Can't tell | Yes | | Can't tell | Yes | | Yes | | | Yes |
|  | 1.4 Is the interpretation of results sufficiently substantiated by data? | Yes | Yes | | Yes | | Yes | Yes | | Yes |  |  | |  | Yes | Yes |  |  | | Yes | | Yes |  | Yes |  | | Yes |  | No | Yes | | Yes | No | | No | | | No |
|  | 1.5 Is there coherence between qualitative data sources, collection, analysis and interpretation? | Yes | Yes | | Yes | | Yes | Yes | | Can't tell |  |  | |  | Yes | Yes |  |  | | Yes | | Yes |  | Yes |  | | Yes |  | Can't tell | Can't tell | | Can't tell | Yes | | Yes | | | Yes |
| Quantitative randomized controlled trials | 2.1 Is randomization appropriately performed? |  |  | |  | |  |  | |  |  |  | |  |  |  |  |  | |  | |  |  |  |  | |  |  |  |  | |  |  | |  | | |  |
|  | 2.2 Are the groups comparable at baseline? |  |  | |  | |  |  | |  |  |  | |  |  |  |  |  | |  | |  |  |  |  | |  |  |  |  | |  |  | |  | | |  |
|  | 2.3 Are there complete outcome data? |  |  | |  | |  |  | |  |  |  | |  |  |  |  |  | |  | |  |  |  |  | |  |  |  |  | |  |  | |  | | |  |
|  | 2.4 Are outcome assessors blinded to the intervention provided? |  |  | |  | |  |  | |  |  |  | |  |  |  |  |  | |  | |  |  |  |  | |  |  |  |  | |  |  | |  | | |  |
|  | 2.5 Did the participants adhere to the assigned intervention? |  |  | |  | |  |  | |  |  |  | |  |  |  |  |  | |  | |  |  |  |  | |  |  |  |  | |  |  | |  | | |  |
| Quantitative non-randomized (non-randomized controlled trials, cohort studies, case-control studies, cross-sectional analytic study) | 3.1 Are the participants representative of the target population? |  |  | |  | |  |  | |  | Can't tell | Yes | | Yes | Can't tell |  | Yes | Yes | |  | |  | Yes |  |  | |  | Yes | Can't tell |  | |  |  | |  | | |  |
|  | 3.2 Are measurements appropriate regarding both the outcome and intervention (or exposure)? |  |  | |  | |  |  | |  | Yes | Yes | | Yes | Yes |  | Yes | Yes | |  | |  | Yes |  |  | |  | No | Yes |  | |  |  | |  | | |  |
|  | 3.3. Are there complete outcome data? |  |  | |  | |  |  | |  | Yes | Yes | | Yes | Can't tell |  | Yes | Yes | |  | |  | Can't tell |  |  | |  | Yes | Yes |  | |  |  | |  | | |  |
|  | 3.4 Are the confounders accounted for in the design and analysis? |  |  | |  | |  |  | |  | Yes | Yes | | Can't tell | No |  | No | Can't tell | |  | |  | Can't tell |  |  | |  | Yes | Can't tell |  | |  |  | |  | | |  |
|  | 3.5 During the study period, is the intervention administered (or exposure occurred) as intended? |  |  | |  | |  |  | |  | Can't tell | Can't tell | | Yes | Can't tell |  | Can't tell | Can't tell | |  | |  | Can't tell |  |  | |  | Yes | Can't tell |  | |  |  | |  | | |  |
| Quantitative descriptive (indicidence or prevalence study without comparison group; opinion survey; case series; case report) | 4.1 Is the sampling strategy relevant to address the research question? |  |  | |  | |  |  | |  |  |  | | Yes |  |  |  |  | |  | |  |  |  | Yes | | Yes |  |  |  | | Yes |  | |  | | |  |
|  | 4.2 Is the sample representative of the target population? |  |  | |  | |  |  | |  |  |  | | Yes |  |  |  |  | |  | |  |  |  | Yes | | Yes |  |  |  | | Yes |  | |  | | |  |
|  | 4.3 Are the measurements appropriate? |  |  | |  | |  |  | |  |  |  | | Yes |  |  |  |  | |  | |  |  |  | Can't tell | | Yes |  |  |  | | Can't tell |  | |  | | |  |
|  | 4.4 Is the risk of nonresponse bias low? |  |  | |  | |  |  | |  |  |  | | No |  |  |  |  | |  | |  |  |  | Can't tell | | Yes |  |  |  | | Can't tell |  | |  | | |  |
|  | 4.5 Is the statistical analysis appropriate to answer the research question? |  |  | |  | |  |  | |  |  |  | | Yes |  |  |  |  | |  | |  |  |  | Yes | | Yes |  |  |  | | Yes |  | |  | | |  |
| Mixed method (also rate the qualitative and quantitative part in addition) | 5.1 Is there an adequate rationale for using a mixed methods design to address the research question? |  |  | |  | |  |  | |  |  |  | |  | Yes |  |  |  | |  | |  |  |  |  | | Yes |  | Yes |  | | Yes |  | |  | | |  |
|  | 5.2 Are the different components of the study effectively integrated to answer the research question? |  |  | |  | |  |  | |  |  |  | |  | Yes |  |  |  | |  | |  |  |  |  | | No |  | No |  | | Yes |  | |  | | |  |
|  | 5.3 Are the outputs of the integration of qualitative and quantitative components adequately interpreted? |  |  | |  | |  |  | |  |  |  | |  | Yes |  |  |  | |  | |  |  |  |  | | No |  | Yes |  | | Yes |  | |  | | |  |
|  | 5.4 Are divergences and inconsistencies between quantitative and qualitative results adequately addressed? |  |  | |  | |  |  | |  |  |  | |  | Yes |  |  |  | |  | |  |  |  |  | | Yes |  | Yes |  | | Yes |  | |  | | |  |
|  | 5.5. Do the different components of the study adhere to the quality criteria of each tradition of the methods involved? |  |  | |  | |  |  | |  |  |  | |  | Yes |  |  |  | |  | |  |  |  |  | | Yes |  | No |  | | No |  | |  | | |  |
