# Supplementary material for: Patients' Health & Well-Being in Inpatient Mental Health-Care Facilities: A Systematic Review
Source: Front Psychiatry. 2022 Jan 3;12:758039. doi: 10.3389/fpsyt.2021.758039 (PMC8761847; doi:10.3389/fpsyt.2021.758039)
Supplement: Supplementary file 2 [file Table_2.DOCX]

Table 2| Results extended

| **Author/year/country /reference no.** | **Study type** | **Target population** | **Participants & sample size** | **Context/inpatient mental health-care facilities** | **Study objectives** | **Methods/Measure** | **Qual** | **Overall conclusions** | **Key findings** | **Building/design feature** | **Domain** | **Outcome** | |
| --- | --- | --- | --- | --- | --- | --- | --- | --- | --- | --- | --- | --- | --- |
| Ben-Zeev et al., 2017, USA (59) | Quantitative non-randomized (cross-sectional) | Patients with high risk of violence (schizophrenia, schizoaffective d., bipolar d., co-occurring substance use d., violence-related incidences) | Patients (schizophrenia, schizoaffective d., bipolar d.) (n=27) | 1 secure state hospital | Identify behavioral & psychosocial correlates of violent ideation & behavior in high-risk violent inpatients with mHealth technology | Speech duration, movement & patient location (via smartphone sensors & beacons, mHealth); Diary study (questionnaires, 6-times/day) | 60% | Delusional beliefs, suicidal ideation, loud ward conditions, negative affect, lack of variability in physical activity, substance related cravings, and some symptoms of withdrawal were linked to violence among individuals with serious mental illness and a history of co-occurring substance use disorders. | Noisy ward conditions were associated with increased odds of violent ideation but not with aggressive behavior. The association remained significant in multivariate models. | Noise | ES | Well-being (violent ideation) | |
| Bowers et al., 2010, UK (83) | Quantitative (descriptive) | Patients (unspecified), staff & visitors | Total (n=1227)  - Patients (n=393)  - Staff (n=638)  - Visitors (n=168) | 61 acute psychiatric wards for adult patients across NHS Trusts in England | Assess acceptability & compare between staff, patients & visitors; Compare opinions reg. other containment measures; Explore acceptability rating by practice of door locking; by patients' age, gender, ethnicity, legally detained or not; by nursing staff qualification & experience | Questionnaire (postal); frequency of ward door being locked; 34 Likert scaled items on acceptability of door locking an acute psychiatric inpatients ward (18 items effect on patients, seven items effect on staff, three items effects on visitors, six items ACMQ) | 60% | Staff should not underrate the significance of locked doors for patients.  Each user group has different perspectives from locked doors.  Staff and visitors tend to have more positive experiences than patients. | Locking' (locked door and wards) was associated with different user group experiences. Staff associated locking with positive, with greater safety benefits and expected patient comfort. Patients saw more adverse effects, were more negative about their stay (in locked ward) and about locking. Patients registered more adverse feelings (depression, anger, worthlessness) than staff or visitors expected. | Locked ward doors | EC  S/A | Mental health (Increased averse feelings: Depression/hopelessness, anger/frustration/irritation, feeling trapped/desperate to escape, worthless/rejected, hinted recovery impacts) | |
| Brooks et al., 1994, USA (93) | Quantitative non-randomized (cross-sectional) | Patients (unspecified) | Measure at ward level (prevalence: bipolar d., manic type, paranoid schizophrenia, psychotic d., adjustment d.) (n=6) | 6 acute care units in a public psychiatric hospital | Determine effects of overcrowding on patients' aggressive behavior | No. seclusion or restraint incidences (1 year); Patient census/unit at or over capacity | 60% | Increased numbers of seclusion and/or restraint incidents are related to overcapacity census. Other patient factors and environmental factors may affect the numbers of seclusion and/or restraint incidents. | A high number of seclusion and/or restraint incidents were related to overcapacity (>28 patients; < 100 square feet / 9 square meter per patient) | Crowding/lack of space; < 100 square feet (9 square meter)/patient | SS | Well-being (aggressive behavior via seclusion and constraint incidences) | |
| Nanda et al., 2011, USA (809) | Mixed-methods, convergent design (quantitative non-randomized & qualitative descriptive) | Patients (unspecified) | Staff (n=22) (patients unspecified, in crisis requiring hospitalization on acute unit) | 1 acute care psychiatric unit, focus: multi purpose lounge | Lay foundation for art use in mental health facilities to reduce anxiety & agitation; Investigate economical ramifications on healthcare organization | PRN medication; Focus groups (staff) | 47% | Nature art significantly reduced the number of incidents or indications of agitated behavior (displaying reduced anxiety and/or aggression) as compared to no art or abstract art. Simple environmental interventions like visual art can reduce medication costs and staff and pharmacy time by providing a visual distraction to alleviate anxiety and agitation in patients. Cost of PRN medication was compared for the different conditions establishing a financial case an annual cost saving of $US4000–27 000, depending on type of art selected. | Significant difference in PRN/patient census ratio between the natural art and control (no art) conditions; significantly lower ratio for realistic nature art than for abstract art and representational art. Nurses perceive that artwork in the patient lounge may have different patient outcomes depending on the type (abstract, abstract representational, nature or none). | Artwork in patient lounge: 1) nature photograph, 2) landscape (abstract-representational), 3) chaotic abstract | ES S/A | Well-being (anxiety, agitation) | |
| Ulrich et al., 2018, Sweden (627) | Quantitative non-randomized (cross-sectional) | Patients (unspecified) | Measurement at hospital level (across all: schizophrenia or other psychosis, bipolar d., personality d., suicide risk) (n=3) | 3 psychiatric hospital (Old, New, Control) | Propose model for ward design to reduce aggression & violence; Evaluate model based on a study of aggressive incidents in psychiatric hospitals (varying design) | No. compulsory injections & restraints | 80% | The proportion of patients requiring injections was significantly reduced in the new hospital (with stress reducing environment) after the move from old hospital (no stress reducing environment). No significant change in number of restraints. | New hospital (9 out of 10 stress-reducing characteristics) resulted in reduction of patient requiring injection and significant reduction of restraints compared to old hospital. | Single rooms, communal areas (spatial, adjust. furniture), low social density, noise reduction, control,  garden, nature views, nature art, daylight, sightlines room-communal areas | SS  ES  EC | Well-being (aggressive behavior) | |
| Bowers et al., 2009, UK (859) | Quantitative non-randomized (cross-sectional) | Patients with acute mental d.s (unspecified) | Measurement at ward level (n=136) | 136 acute psychiatric wards in 67 hospitals within 26 NHS Trusts | Assess relationship of patient violence to: patient characteristics, service and physical environment, patient routines, staff factors, containment, & other patient behaviors | Patient-staff Conflict Checklist (PCC-SR; staff) | 40% | High levels of aggression were associated with a high proportion of patients formally detained under mental health legislation, high patient turnover, alcohol use by patients, ward doors being locked, and higher staffing numbers (especially qualified nurses). | All aggressive behaviors (verbal aggression, physical aggression towards objects, physical aggression towards others) showed a significant relationship to door locking practices and seclusion. | Locked ward doors | EC | Well-being (aggressive behavior, verbal aggression, physical aggression towards objects, physical aggression towards others) | |
| Gallop et al., 1996, Canada (719) | Qualitative (descriptive) | Patients (female) with history of sexual and/or physical abuse | Female patients with sexual and/or physical abuse history (n=18) | 3 psychiatric acute-care facilities; acute-care units of a) large urban teaching hospital; b) urban psychiatric teaching hospital; c) community hospital; mixed-gender | Identify concerns of women with history of abuse reg. safety & hospital experience; Identify desired environmental changes | Semi-structured interviews | 80% | Patients felt unsafe in mixed-gender wards, wished for segregation, had concerns regarding night time issues (lights, doors, routines checks and staff availability); the primary patient-nurse relationship to guarantee consistency of care was associated with sense of safety, comfort, and empowerment. Participants suggested environmental changes to limit their exposure to male patients, to provide them with more options in managing night time difficulties, and to facilitate their engagement in treatment planning and the therapeutic relationship. Feelings of safety and control afforded by the physical, social and procedural environment were trauma-related. | Mixed-gender wards associated with feeling unsafe, threat of violence and sexual assault. At night-time there is a wish for softer lights left on to afford visibility, rather than the use of flashlights during routine checks. Some preferred bedroom doors to be kept open at night, others preferred them shut. | Single vs mixed-gender wards, soft lights at night (oppose to flashlight use), closed bedroom doors | SS  EC | Mental health (trauma-related safety & control feelings) | |
| Johnson & Delaney, 2006, USA (881) | Qualitative (grounded theory) | Patients (unspecified) | Patients (depression, schizophrenia, schizoaffective d., bipolar affective d.) (n=12), Staff (n=16) | 2 adult locked inpatient psychiatric units (general psychiatric; capacity > 15 beds, short-term stay); 2 medium-to-large not-for-profit medical centers | Develop theory of patient violence prevention; Describe context & conditions of used violence interventions | Observations; Formal interviews (patient & staff) | 100% | Social process framework was developed ('Keeping the Unit Safe') that acts on four dimensions to prevent patient violence: ideology, people, space, and time. | The organization and structure of the unit are related to the prevention of violence. Lower violence-risks was associated with visibility through ward design, placement nursing rooms, reduction of hidden spaces, and clear boundaries of prohibited spaces. Crowding and lack of personal space were associated by patient with risk of violent behavior. | Crowding, personal space/common areas of adequate size, visibility (ward design & location nursing rooms), rules managing spaces & people accessibility (incl. personal space & territoriality), tangible boundaries (locked doors, closed-off areas e.g., kitchen) | SS  EC | Well-being (Aggressive behavior) | |
| Lindgren et al., 2015, Sweden (838) | Qualitative (phenomenological) | Patients (female) who self-harm | Female patients who self-harm (n=6) | 2 locked acute psychiatric wards (mixed-gender & diagnoses) | Describe features of everyday life in psychiatric inpatient care experienced by women who self-harm | Observations; Informal interviews | 100% | Main feature of everyday life: ‘being surrounded by disorder’, which consisted of ‘living in a confusing environment, being subject to routines and rules that offer safety but lack consistency’ and ‘waiting both in loneliness and in togetherness’. Nursing staff spent minimal time with the patients; women turned to each other for support, care and companionship. | Confusing environment: wards overcrowded; doors were locked; noisy events creating non-soothing atmosphere; patients' beds sometimes in corridors or meeting rooms and frequently moved from one area to another; loud and frequent sounds day and night; night sounds caused sleep disruption, panic, and a desire to escape; being witnesses of violent behavior caused distress. Overall positioned as non-conducive to recovery. | Crowding, locked ward doors, beds not in room & frequently moved, noise | SS  ES  EC | Mental health (trauma-related feelings of confusion and distress, states of panic, wish to escape, sleep disruption) | |
| Beauchemin & Hays, 1996, Canada (51) | Quantitative non-randomized (quasi-experimental) | Patients with depression | Patients with depression (major depressive d. single/recurrent, bipolar, depression N.O.S.) (n=174) | 2 psychiatric wards of one hospital | Determine if amount of sunlight in patient rooms had impact hospitalization length | Admissions records (2 years) | 60% | The amount of natural lighting provided in a hospital room seemed to affect the duration of a patient's stay, with shorter stays for those staying in a room with increased natural light (bright) and longer stays for those staying in a room with decreased natural light (dim). | Patients in brightly lit rooms had a mean 2.6-day shorter hospitalization than patients in dimly lit rooms, a difference more marked in men (6.8 days) than in women (0.7 days) | Natural light in patient rooms, either bright (max. 5000 lux) or dim (max. 300 lux) | ES | Recovery (length of stay) | |
| Holmes et al., 2004, Canada (267) | Qualitative (phenomenological) | Patients (unspecified) | Patients with psychotic d.s (n= 6) | 1 specialized psychiatric care unit in psychiatric hospital (25 beds, mixed gender & diagnoses) | Describe daily experiences of patients to help improve the quality of nursing care | In-depth interviews | 100% | Patients experienced intensified negative emotions during seclusion periods which added to existing feelings of exclusion, rejection, abandonment, and isolation. The seclusion from nurses worsens the situation and negative behavior is motivated by a need to connect with staff. | Patients describe an intensification of negative emotions when being isolated and negative perceptions of isolation, and how they cope with this. Negative emotions are associated with seclusion room experiences. | Seclusion room | SS  EC  S/A | Well-being (feelings of exclusion, rejection, abandonment, anger, fear, shame, humiliation, sadness, depressive feelings) | |
| Maloret & Scott, 2018, UK (367) | Qualitative (phenomenological) | Patients with autistic spectrum condition (ASC) | Former psychiatric inpatients with ASC diagnosis (co-diagnoses: anxiety, psychotic, mood d., depression, eating d., addiction) (=20) | Unspecified acute mental facilities | Explore ASC inpatients experience | Semi-structured interviews | 80% | Sensory aspects of the physical environment caused anxiety which was often overlooked by mental health practitioners. Greater attention to anxiety-inducing environments is needed when planning for improvements and more research is needed to understand experiences. | Specific sensory aspects of the physical environment caused anxiety and were perceived as problematic: excessively bright lighting, overpowering smell of strong cleaning products, continually loud air conditioning system, and taste, smell and texture of the hospital food. This lead to anxiety and coping strategies, which involved self-harm and violent responses for some. There was also a need to isolate to cope with rising anxiety levels, but this was not always possible. Staff can perceive the highlighted issues that impact the mental well-being of inpatients as small/trivial. With greater insight, practitioner responses could be more sensitive and understanding. | Bright lighting, air conditioning & other noise, strong smells (cleaning products), need for quiet and solitude space | ES | Mental health (anxiety and related coping strategies (e.g., aggression, self-harm, social withdrawal)) | |
| O'Brien & Cole, 2004, Australia (354) | Mixed-methods, convergent design (quantitative descriptive & qualitative phenomenological) | Patients requiring close observation (e.g. suicidal patients, not specified) | Patients (who had been cared for in the close observation area, unspecified), relatives, staff (n=42) | 1 eight-bed close observation area within acute inpatient general hospital psychiatric facility | Develop understanding of context & experiences of nurses, patients, and relatives in close-observation area in acute psychiatric facility; Develop recommendations for clinical practice guidelines | No. seclusion incidences & PRN medication use (1 mth); Security use (5 mths); Interviews & focus groups (patients, relatives, staff) | 80% | Problems in eight-bed close observation area: High rates of seclusion, paucity of documentation related to outcomes of interventions and therapeutic interventions when used, lack of explanation or education of patients and/or relatives about seclusion and prn medication, poor standard of documentation of critical issues. There was a high level of agreement across participants about design and environmental problems and the problems associated with lack of activity and structure. Mental health nursing care is the most important intervention in the area of close-observation care and mental health nursing needs to develop clinical practice guidelines specific to this area of nursing. | In relation to the design and environment, problems included: prison-like atmosphere and lack of privacy, security, personal space, and environmental control to withdrawal, associated with the fish-bowl design, no doors on some bedrooms, no curtains on windows. Witnessing seclusion incidences was “traumatizing”. Poor levels of comfort, with insufficient bathrooms and toilets, insufficient recreational spaces**,** lack of private areas for visitors and nurse counselling activities, no direct access to meal reheating or beverage-making facilities, and no staff handwashing facilities. | Eight-bed close observation area in fish-bowl design (lack of privacy and doors, no environmental withdrawal possibilities given shared room), mixed-gender ward, prison-like atmosphere, poor environmental conditions (bathroom, toilets) and little comfort | SS  ES  EC  S/A | Well-being (feeling unsafe, discomfort, feeling traumatized) | |
| Benedetti et al., 2001, Italy (209) | Quantitative non-randomized (quasi-experimental) | Patients with depression | Depressed patients (n=602) - Unipolar (n=415)  - Bipolar (n=187) | 1 psychiatric ward | Compare hospitalization length of depressed patients exposed to morning sunlight with those exposed to evening sunlight | Admission charts (3 years) | 80% | Natural sunlight can be an underestimated and uncontrolled light therapy for bipolardepression. Exposure to morning sunlight can have therapeutic effects in bipolar depression. | There were no significant differences associated to room lighting condition for unipolar patients; bipolar depressed inpatients exposed to natural sunlight in the morning had a shorter hospital stay than patients exposed in the evening. | Sunlight in patient rooms, either morning (max. 15,500 lux) or evening (max. 3000 lux) | ES | Recovery (length of stay) | |
| Donald et al., 2015, Australia (525) | Qualitative (thematic analysis) | Patients (unspecified) | Patients (unspecified, n=19) | 2 wards (28 beds each; 1 Low Dependency Unit, 1 secure High Dependency Unit) at 1 large city hospital | Assess role of psychiatric settings in recovery; Examine everyday experience of patients to understand influences of environmental aspects on recovery | Semi-structured interviews (n=9); Focus groups (n=10) | 60% | Analysis identified three major themes concerning consumers’ experience within the unit: 1) the importance of staff; 2) lack of clear architectural identity resulting in confused or confusing space; 3) limited amenity due to poor architectural design. A collective care environment should prioritize patient-staff and patient-patient interactions and promote architectural improvements, such as views to greenery and community space to counteract boredom and isolation. | Staff-patient interactions play an important role in the perception of the social environment as comfortable and safe (staff approachable, affording personal control); low stimulation ("sterile environment") and lack of privacy as of glass walls in treatment rooms create confusing environment (no cues); lack of amenities causes feeling of being trapped. | Lack of privacy in glass treatment rooms, sterile (low stimulation) environment, lack of activity amenities | SS  ES | Well-being (spatial confusion, boredom, feeling trapped, need for distraction) | |
| Edwards & Hults, 1970, USA (280) | Mixed-methods, convergent design (quantitative non-randomized / descriptive analysis & qualitative descriptive/phenomenological) | Patients (unspecified) & staff | Staff (n=26)  Patients (n=8) | 2 psychiatric wards (one "open", one "closed") in a veterans hospital | Study effect of nursing station open vs closed design (glass/no glass window glass) on patient behavior, staff-patient communication, and staff & patient preferences (glass/no glass) | No. patient interaction (time study); Questionnaires (patients & staff); In-depth interviews (patients); Clinical observations | 67% | After the removal of the window glass from the nursing station, staff members spent less time in the office and more time in the dayroom interacting with the patients, so patients came to the office less often. The removal of the barrier to communication encouraged more meaningful interactions and a more relaxed ward milieu. | After opening the nursing station by glass removal, patients visited it less often and staff spent less time in the station. The number of patient-initiated interactions decreased significantly as staff appeared to be more accessible. Patients (88%) and staff (84%) preferred the open station design. Fewer patients felt they were interrupting staff and fewer were bothered by seeing staff members talking and laughing. Patients no longer felt the staff needed protection from them, that they were not a threat. Shift from indirect, nonverbal communication to direct, verbal communication. Better contact between staff and patients in the dayroom and corridor and patients no longer felt shut out. More patient input to change-of-staff report. Staff have better ventilation. | Closed vs opened nursing station (removal of window glass) | S/A | Well-being (better verbal communication with staff, patient needs are better met, feeling less bothersome & threatening) | |
| Haglund & von Essen, 2005, Sweden (259) | Qualitative (descriptive) | Patients (unspecified) | Patients (voluntary admitted; common diagnoses: mood d., schizophrenia, other psychotic d.s, anxiety, personality d.) (n=20) | 7 inpatient wards | Describe voluntarily admitted patients' perceptions of advantages & disadvantages about care on ward with locked entrance door; Underlying aim: study if patients perceived coercion on such wards | Semi-structured interviews | 80% | Patients perceive a variety of advantages and disadvantages, for themselves, their visitors and staff, connected to locked entrance doors at psychiatric wards. A locked door may make the ward appear as both a prison and a sanctuary. It is important that staff try to minimize the patients' concerns connected to locked doors. | Patients perceived a variety of advantages and disadvantages, for themselves, their visitors and staff. Perceived advantages included: a feeling of protection from the outside; the possibility of controlling patients, implying calmness and safety for staff and patients; helping staff provide secure and efficient care; a sense of safety; relief for relatives; more time for patients. Perceived disadvantages: confinement; a feeling of dependence on staff; feeling worse emotionally/extreme emotional distress incl. suicidal thoughts; extra work for staff to open the locked door; a non-caring environment, like a prison; patients become passive as they avoid outdoor activities; staff have the power; concerns that visitors may feel unwelcome and insecure; concerns for visitors' reactions; frustration; adaptation to other patients' needs. | Locked ward doors | EC S/A | Mental health (significant emotional distress and symptoms (state of panic, suicidal thoughts, nervousness, depression, fearfulness, anger), feeling dependent, decreased self-confidence, passiveness, feeling safe from the outside) | |
| Kulkarni et al., 2014, Australia (713) | Quantitative non-randomized (cross-sectional) | Patients (female, unspecified) | Female patients (psychotic, mood d., post-partum psychosis/depr., anxiety d., eating d., personality d.) (n=65)  - intervention (n=44)  - control (n=21) Staff (n=20) | 2 psychiatric wards in a hospital | Assess impact of female-only area within a mixed-gender facility on female patients' safety (objective & perceived) and experience of care | Safety incidents reports (6 mths); Questionnaire (patients & staff) | 80% | Establishing female-only areas in psychiatry wards is an effective way to improve the safety and experience of care for female patients. | Compared with a mixed gender ward, a similar ward comprising a female-only area was associated with substantially fewer documented incidents compromising the safety of female patients, and resulted in a more positive perception of safety and experience of care. While the number of incidents overall was too small for a meaningful statistical analysis, the six-fold difference in the raw data was striking and suggestive of a significantly safer service model. Staff feedback also endorsed the introduction of a female only area. | Female-only area | EC | Well-being (perceived safety and experience of care, satisfaction, comfort) | |
| Lamanna et al., 2016, Canada (726) | Qualitative (interpretive theoretical framework) | Patients (unspecified) & staff | Patients (psychotic d., mood d., other) (n=14) Staff (n=10) | 1 unit in psychiatric department (acute, intermediate and general  sections) of general hospital | Explore & compare patient and staff perspectives on factors affecting patient aggression (verbal & physical) | Semi-structured interviews | 80% | Aggression is perceived to have a wide range of origins spanning personal experiences and organizational policies, suggesting that a wide range of prevention strategies are needed. Illness may contribute to aggression through additional pathways, including patients’ stress and reduced emotional control. Personal and organizational factors (confinement, restrictions, lack of engagement with staff) were associated to aggressive incidents. | Patient views: physical/spatial confinement was associated with aggression (anger); coping strategies utilized in the community (such as listening to music) were not available in the hospital. Clinicians views: patient confinement leads to a loss of autonomy and feelings of powerlessness, leading in turn to aggressive incidents. This process reinforces itself, where physical confinement can cause fear or tension leading to aggressive behavior, leading to caution from staff at allowing patient out of the unit. Patient & clinician views: lack of access to personal possessions, restrictions on activities and boredom results in anger and frustrations (amplified by physical confinement). | Spatial confinement (if hospitalized involuntarily, secluded in their rooms, denied passes off the unit, or kept to scheduled passes) | EC S/A | Well-being (aggressive behavior fostered by feeling trapped, losing autonomy) | |
| Muir-Cochrane et al., 2013, Australia (406) | Qualitative (phenomenological) | Patients held involuntarily, absconding experience/attempt (unspecified) | Former psychiatric inpatients, involuntarily admitted with absconding experience (n=12) | Unspecified inpatient psychiatric unit | Explore experiences of involuntarily held patients with absconding experience/attempt in an inpatient psychiatric unit | Semi-structured interviews | 80% | Inpatient psychiatric unit was experienced by consumers as both a safe (environment was perceived as a sanctuary and therapeutic place) and an unsafe place (negative associations and interactions with mental condition). Triggers for absconding; individual factors, social factors, physical environment and symbolic environment. The main reason consumers absconded was feeling unsafe in the hospital environment. | Physical environment factors associated with absconding include; hospital crowding, excess noise, too busy, poor thermal comfort, and ugly/prison like. Calming surroundings (natural surroundings outdoors and the use of color indoors) were associated with healing. Contrasting perceptions of privacy in relation to safety include; too much privacy associated with opportunities to abscond, private settings associated with sanctuary spaces. Mixed-gender communal spaces were perceived as unsafe by women. Separated nurse station was perceived as determinant of power dynamics in the ward. Lack of freedom was associated with a prison-like environment. Unfamiliarity with settings trigger associations of a frightening and uncomfortable space. Facilities not promoting autonomy related to reduced control. | Crowding, noise, temperature discomfort, unpleasant aesthetics, calming surroundings (naturalness, color indoors), familiar/unfamiliar prison-like associations, mixed-gender settings; separate nurse station; facilities not promoting autonomy | SS  ES  EC  S/A | Well-being (absconding behavior, comfort/discomfort, feelings of safety, healing association, boredom, lack of autonomy, psychological distance to staff) | |
| Smith & Jones, 2014, UK (546) | Mixed-methods, sequential explanatory design (quantitative non-randomized / descriptive analysis; qualitative phenomenological) | Patients in PICU (acute disturbed phase, high risk to self/other safety, unspecified) & staff | PICU Patients (male, seclusion and sensory room experience, pathology not specified) (n=7) Staff (n=10) | 1 male-only Psychiatric Intensive Care Unit (PICU), "locked", for compulsorily detained patients, with 15 beds in an mental health facility | Identify if sensory room introduction reduced seclusion rates; Explore staff & patients’ experiences of using sensory room | No. seclusion incidences (3 mths pre & post intervention); semi-structured interviews (13 mths post intervention) | 67% | The use of a sensory room improved both staff and patients’ experience of the ICU and should be an intervention considered by other ICUs and inpatient psychiatric settings. But the seclusion rates did not decrease in the first three months following the introduction of the sensory room on the PICU. | The effects of a new sensory room for the PICU were contradicting. While the qualitative data indicated increased amount and length of incidents at the ward, the perceptions by patients and staff were positive, in terms of overall PICU experience and communication. Both staff and patients felt the sensory room has reduced symptoms. The difference may be explained; staff perceived the room worked to de-escalate patients, except for highly aggressive patients; and few patients were responsible for many incidents. | Sensory room with equipment | ES | Mental health (perceived reduction in symptoms (staff and patients), calming and aiding de-escalation, relaxing and stress reducing, socialization, increased communication) | |
| Van Wijk et al., 2014, South Africa (857) | Qualitative (phenomenological) | Patients (unspecified) | Patients (not psychotic; n=40; n=20 each site) | 2 pre-discharge "open" wards at each of 2 state mental health facilities | Describe patients’ perceptions of environmental & staff factors that contribute to their aggressive behavior at mental health facility; Propose prevention and management strategies | Semi-structured interviews | 100% | Patients were disturbed by the unpleasant atmosphere in the ward and their perceptions revealed that many environmental and situational factors contribute to frustration and a reduced threshold to aggressive outbursts and disruptive, violent behavior against self, fellow patients and/or staff members. The significant poor institutional condition and procedures (physical abuse by staff) created an environment non-conducive to recovery. | Contributors to aggressive behavior and significant emotional distress: Unhygienic conditions in the ward, noise levels, crowding, shortage of personal hygiene items (such as face cloths, towels, toilet paper, deodorants, razors and toothbrushes), patients with different diagnoses in one ward, safety issues (stealing), witnessing seclusion incidences frightening, experience of physical abuse by staff, increased vulnerability. | Crowding, noise, unhygienic conditions, seclusions rooms, mixed-pathology ward | SS  ES  EC  S/A | Well-being (aggressive behavior, emotional distress) | |
| Wood & Pistrang, 2004, UK (977) | Qualitative (phenomenological) | Patients (unspecified) | Patients (bipolar affective d., depression, schizophrenia, borderline p. d.) (n=9) Staff (n=7) | 1 acute mental health unit with 4 wards in capital, 1 ward selected for the study | Exploring the experience of being a psychiatric inpatient, specifically reg. safety & threat feelings | Semi-structured interviews (patient & staff) | 100% | There were 10 themes, grouped into three clusters—patient interactions, staff behavior and attitudes, and non-consensual treatment—that described factors impacting on feelings of safety. Patients’ accounts were characterized by an overwhelming sense of vulnerability and helplessness. Staff accounts were largely consistent with patients’ accounts. | Lack of privacy as of shared bedrooms contributed to a heightened sense of unsafety; social environment (encounters with patients and staff) contributed to feeling unsafe; patients interaction in mixed-gender settings were associated with feeling unsafe. Use of seclusion room was frightening and threatening. | Mixed-gender wards, shared bedrooms, seclusion rooms | SS  EC  S/A | Well-being (feeling unsafe, vulnerable, threatened) | |
| Connellan K., Riggs D. and Due C., 2015 (Australia) | Qualitative (phenomenological) | Patients (unspecified) & staff | Patients and staff (n=unspecified) | 1 large mental health ward in Australia, containing 2 main wards | Explore what the effect of glass is on the users | Ethnographic observations based on 34 hours of observation at morning and afternoon over a 10 week period | 60% | Glass is a complex material that can be engineered to perform complex tasks (such as bringing different acoustic effects based on the glass and how or where it is used, it can ‘miscommunicate, beguile, and tease’), other than merely to be used as a lightweight building material and conduit of sunlight. | Glass can give the appearance of visibility, safety and openness but can also bring a perceptible barrier to close physical communication.  Glass reflections can be distracting/creating busyness/duplication, ambiguous space that is hard orientate in; one patient was reported to be mesmerized and entertained by the glass reflections. | Glass ration interior design (duty station and across ward), glass ratio interior design, glass | ES  EC  S/A | Well-being (actual and sense of safety, mesmerizing, distraction, confusion, lack of orientation) | |
| Due C., Connellan K. and Riggs D., 2012 (Australia) | Qualitative (phenomenological) | Patients (unspecified) & staff | Patients and staff (n=unspecified) | 1 large mental health ward in Australia, containing 2 main wards, focus here high depend unit (HDU) and open wards | Discuss the relationship between surveillance techniques, security measures and patient violence in mental health wards | Ethnographic observations based on 34 hours of observation at morning and afternoon over a 10 week period | 60% | There is a suggested relationship between security measures (as of loss of control) and aggression. There needs to be close attention paid to the ways in which forms of surveillance exacerbate rather than prevent the need for security measures. | Acts of violence are typically preceded by an incident within the unit that was related to the implementation of security measures aimed at controlling non-compliant behavior; this can be categorized as “loss of control”; Presence of security personnel to control non-compliant behavior created a frightening (patients laid themselves on the ground) and tense atmosphere. Control over patient’s day-to-day functioning (availability of food and drink) in HDU reduced. Loss of control over own belongings elicited aggressive behavior. Patients seem to prefer passive forms of observation (CCTV) oppose to direct observation (spending time around duty station). | CCTV cameras as passive form of observation, availability/access to day-to-day facilities (food and drink), no access to personal belongings | EC | Well-being (aggressive behavior, being frightened and disturbed, comfortable/uncomfortable) | |
| Riggs D., Due C. and Connellan K., 2013 (Australia) | Qualitative (phenomenological) | Patients (unspecified) & staff | Patients and staff (n=unspecified) | 1 large mental health ward in Australia, containing 2 main wards, focus here high depend unit (HDU) and open wards | Explore the role and use of a duty station in relation to both staff and patients; focus on power differentials. | Ethnographic observations based on 34 hours of observation at morning and afternoon over a 10 week period | 60% | The findings of the duty station as problematic leads to the paper questioning the promotion of psychological wellbeing in mental health wards if duty stations are used solely to reinforce power differentials between patients and staff in ways that contribute to a physical gap between the two groups. | The duty station often functioned in a problematic way for surveillance and administration; this was due to its location, glass frame, and the use of the duty station as a means of control for staff. Comparison of two duty station designs indicated the different impact on staff-patient interaction. | Different designs of the nursing station (ratio of Glass, open/closed panel), CCTV; ledger; door on the side | S/A | Well-being (psychological distance between staff and patients/rehabilitative interaction, communication, feeling overly scrutinized) | |
|  | | | | | | | | | | | | |  |
